# Real‐Time Digital Micromotor Tracking‐Enabled Ultrasensitive Immunoassay

**DOI:** 10.1002/advs.202521243

**Published:** 2026-02-21

**Authors:** Jingjing Shi, Zuhua Yu, Hui Tian, Wenjiao Fan, Yuanyuan Sun, Wei Ren, Chenghui Liu

**Affiliations:** ^1^ School of Chemistry & Chemical Engineering Shaanxi Normal University Xi'an P. R. China; ^2^ School of Cyber Science and Engineering Zhengzhou University Zhengzhou P. R. China; ^3^ Department of Translational Medicine Center The First Affiliated Hospital of Zhengzhou University Zhengzhou P. R. China

**Keywords:** artificial intelligence, digital biosensing, immunoassay, micromotor, trajectory tracking

## Abstract

Digital bioassays are emerging as a pivotal technology in disease prevention and diagnostics due to their single‐molecule level detection capability. However, elaborate microchamber fabrication, high‐end equipment, and skilled manipulation are required to enable the end ‐ point digital signal readout. Herein, we propose a novel concept of artificial intelligence (AI)‐facilitated real‐time digital micromotor tracking‐enabled immunoassay (AI‐dMIA), which leverages a self‐developed multi‐microparticle tracking system to monitor micromotor motion trajectories in a real‐time manner for digital protein analysis. In this design, even a single molecule‐bridged immunobinding event on the fully‐open microparticle's surface can induce obvious motion behaviors and trajectories, forming a positive micromotor that can be accurately tracked and discriminated from the negative ones in real time by a bright‐field microscope integrated with the AI algorithm. The AI‐dMIA gets rid of the complex process of microchamber fabrication and fluorescence signal yielding/amplification to generate digital counting events. It also exhibits powerful processing capacity to simultaneously track up to thousands of motor trajectories on a large scale, no longer requiring the high‐end equipment that is essential to traditional end‐point digital bioassays. The AI‐dMIA not only demonstrates a robust immunosensing approach but also provides a new alternative for developing next‐generation digital micromotor‐based platforms.

## Graphical abstract

An AI‐facilitated real‐time digital micromotor tracking‐enabled immunoassay is developed, which leverages a self‐developed multi‐microparticle tracking system to monitor micromotor motion trajectories in a real‐time manner for digital protein analysis. The AI‐assisted micromotor digital immunoassay exhibits a powerful processing capacity to simultaneously track up to thousands of motor trajectories, which paves a new path for developing next‐generation micromotor‐based digital bioassays.

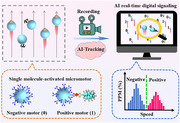

## 1 Introduction

Digital bioassays have evolved into a shining star in ultrasensitive analysis of ultralow‐abundance biomarkers toward early diagnosis and effective therapeutic interventions of diseases [1, 2, 3, 4]. Completely different from conventional bulk measurement‐based analog signal readout technologies, in a classical digital bioassay, the trace target molecules are individually compartmentalized into numerous fL∼nL level sealed microchambers (e.g., microwells or droplets) that act as independent reaction units, each of which contains either 0 or 1 target molecule obeying the Poisson distribution. The microchambers loaded with a single target molecule will actuate signal generation and be lit up, and recorded as positive (1), whereas the target‐free ones will be read as negative (0). Thus, target quantification can be achieved by digitally counting the positive/negative microchambers [5, 6, 7, 8, 9, 10]. Due to the unique binary counting signal readout mode, digital bioassays, represented by commercial single molecule array (Simoa) and droplet digital polymerase chain reaction (ddPCR), possess single‐molecule level sensitivity, breaking through the inherent sensitivity limitations in analog signal detection technologies [11, 12, 13, 14, 15]. Though powerful and promising, the current digital bioassays generally suffer from some intrinsic restrictions. For instance, they are highly dependent on uniform sealed microchambers for physical partition of target molecules, which compels proprietary and elaborate microfabrication consumables, proficient operators, and expensive high‐end equipment for distinguishing positive/negative signals, confining their implementations only to central laboratories [16, 17, 18, 19, 20, 21]. What is more, the prevalent digital bioassays as well as some pioneering non‐sealed microbead‐based digital strategies overly rely on end‐point fluorescence detection [22, 23, 24, 25], which necessitates the use of enzyme‐catalyzed or nucleic acid signal amplification reactions to guarantee the yield of sufficient fluorescence signals by a single target molecule for the discrimination of positive/negative events. Moreover, for the microbead‐based digital bioassays, the fluorescence signal generated by a single immunocomplex is typically confined to a small localized region on the bead surface, which will inevitably cause partial positive signals to fall outside the focal plane during fluorescence imaging or flow cytometer scanning, thereby compromising the overall detection efficiency [26].

Different from the existing digital bioassays, herein, by using protein biomarkers as model analytes, we propose a new generation of artificial intelligence (**AI**)‐facilitated real‐time **d** igital **m** icromotor tracking‐enabled **i** mmuno **a** ssay (AI‐dMIA), which has several unique advantages to guarantee robust binary digital signaling. On the one hand, different from conventional multisite‐driven micromotors [27, 28, 29, 30, 31, 32], in the AI‐dMIA, the magnetic driving power is introduced by a single‐molecule binding event on the confined target‐payload site of the micromotor, which can induce distinct motion trajectories that are employed to determine digital positive signals in a real‐time manner. On the other hand, a multi‐microparticle tracking system (MTS) that possesses powerful processing capacity to simultaneously track up to thousands of micromotors on a large scale has been developed to record and analyze the motion trajectories. Thus, the single target molecule binding‐actuated magnetic micromotors can be precisely tracked in real time and easily identified as positive motors by AI algorithms from a large pool of negative ones. This AI‐dMIA platform does not need sophisticated microfabrication or high‐end equipment, opening an appealing way for fabricating new conceptual real‐time digital immunoassays.

## 2 Results and Discussion

### 2.1 Design Principle of the AI‐dMIA

**FIGURE 1:**
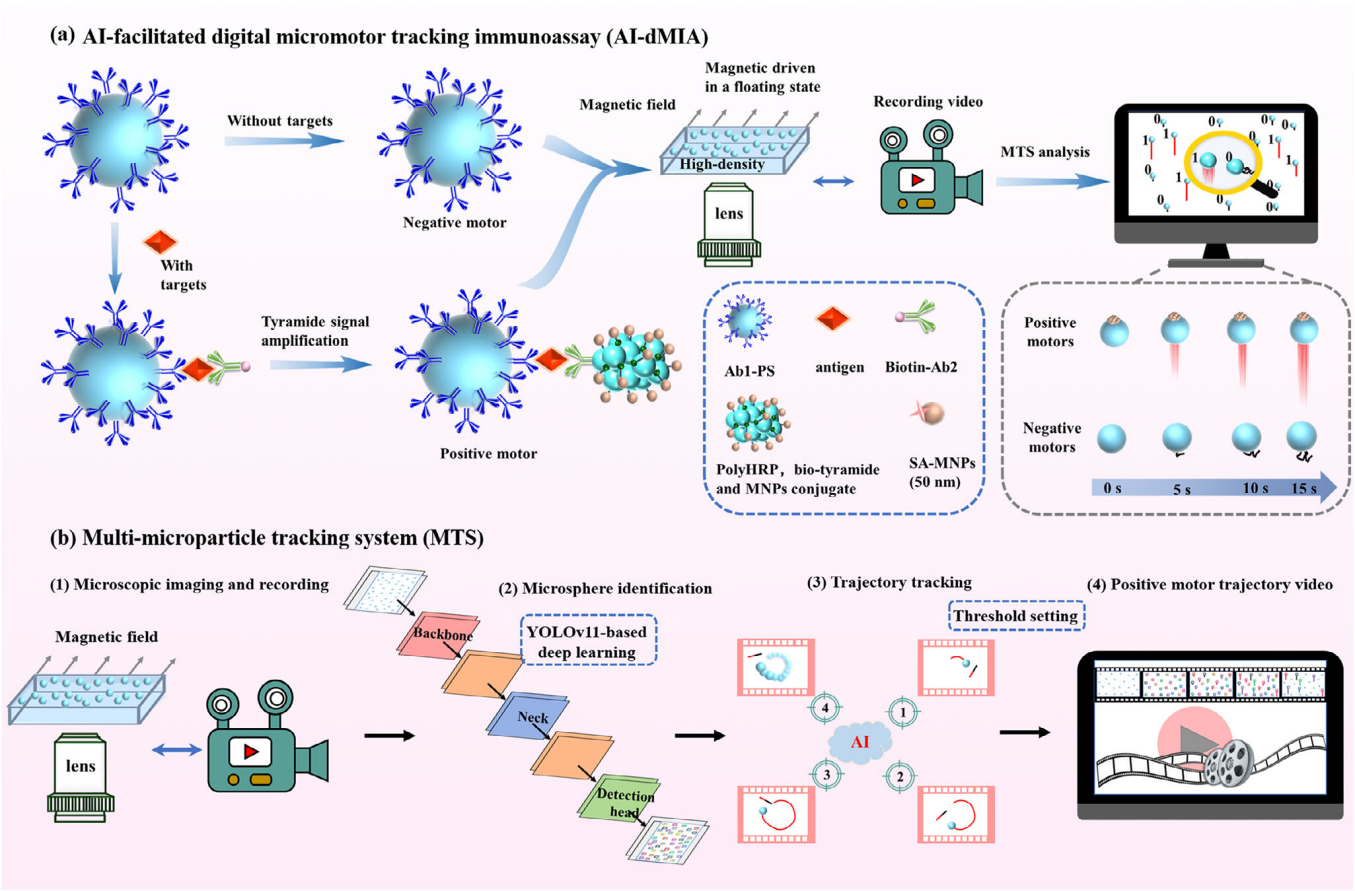
(a) Schematic illustration of the AI‐dMIA design principle for protein analysis. Illustration of motion trajectories induced by positive and negative motors in different periods. (b) Streamline of the MTS system, which includes microscopic imaging and recording, microsphere identification, trajectory tracking, and positive motor trajectory video forming.

Exemplified by magnetic driving, a novel concept of AI‐dMIA is proposed, which utilizes AI algorithms to track single‐molecule binding‐actuated micromotor trajectories for the digital quantification of protein biomarkers. As illustrated in Figure 1a, polystyrene microspheres (6 µm in diameter, denoted as PS_6_) functionalized with capture antibodies (Ab1‐PS_6_) serve as fully open carriers to capture target molecules. When the amount of target proteins is much less than that of PS_6_, only 1 or 0 protein molecule will be captured on each PS_6_ following the Poisson distribution. The PS_6_ loaded with one protein molecule will undergo a sandwich immunoreaction with biotin‐labeled detection antibody (bio‐Ab2). Afterward, streptavidin‐polyHRP (SA‐polyHRP) is introduced to catalytically deposit a large amount of biotin‐tyramine (bio‐tyramine) through tyramide signal amplification (TSA) on the target protein‐carrying PS_6_, which can capture numerous SA‐labeled magnetic nanoparticles (50 nm in diameter, denoted as MNPs_50_) to the PS_6_, forming PS_6_‐MNPs_50_ magnetic micromotors. Conversely, target‐free PS_6_ will not be deposited with MNPs_50_ (MNPs_50_‐free PS_6_). Then, all the PS_6_‐MNPs_50_ and the MNPs_50_‐free PS_6_ are immersed in a testing liquid medium that has a density higher than that of the PS_6_ to ensure that PS_6_ remains floating, thus greatly reducing their moving resistance. Being exposed to a fixed external magnetic field, all motion trajectories of PS_6_ can be easily recorded by using a common bright‐field microscope, followed by using our MTS to undergo the large‐scale real‐time trajectory tracking analysis. Accordingly, the single molecule immunoreaction‐induced PS_6_‐MNPs_50_ will exhibit obvious motion along the magnetic field direction and thus be identified by the MTS as positive motors, whereas MNPs_50_‐free PS_6_ displaying only irregular Brownian motion in place will be signed as negative. Thus, the accurate identification and digital counting of positive motors can be automatically fulfilled for target protein quantification by the MTS, whose workflow is vividly illustrated in Figure 1b. Briefly, the recorded motion video is segmented to precisely identify PS_6_ by using YOLOv11‐based deep learning, and then the percentage of positive motors (PPM) is determined by tracking PS_6_ trajectories and setting a threshold.

### 2.2 Evaluating the Performance of MTS in Multi‐Microparticle Identification and Trajectory Tracking

**FIGURE 2:**
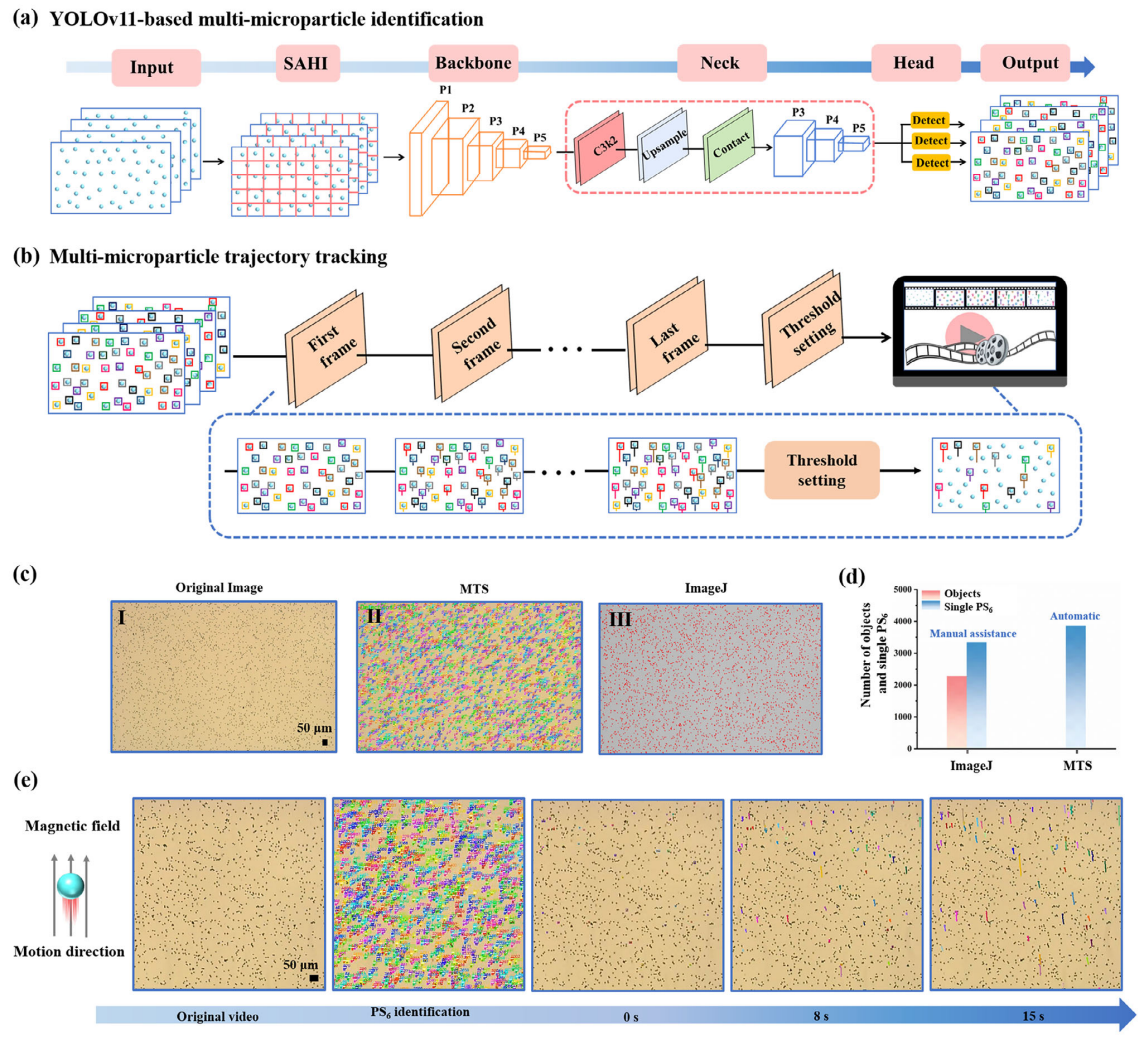
Evaluating the performance of MTS in multi‐microparticle identification and trajectory tracking. (a) Algorithm framework of multi‐microparticle identification based on YOLOv11. (b) Simple algorithm framework for the trajectory tracking of MTS. (c) Comparing the counting capabilities of ImageJ and MTS: (I) Original image captured by a bright‐field microscope; (II) Identifying PS_6_ by MTS; (III) Recognizing PS_6_ by using ImageJ. (d) Comparison of the total number of objects and single PS_6_ processed by ImageJ and MTS, respectively. (e) The identification of total PS_6_ and the trajectory length of positive motors in different periods were analyzed by the MTS, where the motion direction is upward along the magnetic field direction. Scale bar: 50 µm.

Accurate real‐time micromotor tracking is the foundation of the AI‐dMIA concept. The self‐programmed MTS system integrates multi‐microparticle identification and trajectory tracking into a streamlined all‐in‐one workflow, which can well address the challenge of simultaneously tracking thousands of trajectories on a large scale. The detailed algorithm framework of the MTS is displayed in Figure 2a,b. Specifically, motion videos are segmented into numerous overlapping image blocks via slicing aided hyper inference (SAHI), which combines with YOLOv11 for object identification (Figure 2a). The Otsu adaptive threshold segmentation quantifies object pixel coverage, after which the number of single PS_6_ per object is evaluated using kernel density estimation (KDE) statistics based on the single PS_6_ area, thereby achieving accurate counts. Afterward, the simple online and real‐time tracking (SORT) algorithm processes the identification results of consecutive frames to enable cross‐frame association, thereby supporting real‐time, large‐scale trajectory tracking of thousands of objects (Figure 2b). Finally, the number of positive motors, PPM, and a trajectory‐marked video comprising only positive motors are obtained by setting a threshold.

Before the model training, an initial dataset of 1210 frame images is divided into the training set, the validation set, and the testing set in a ratio of 7:2:1 after annotation. The trained YOLOv11 model exhibits high identification precision, with an average precision reaching 97.76% (Table S1). To further validate the overall performance of the MTS, we tested the complete video sequences with manually correlated trajectories, achieving a 92.72% MOTA and confirming its tracking effectiveness (Table S2). Additionally, the counting capacity of MTS is compared with ImageJ, which demonstrates a superior capability in terms of single PS_6_ resolution counting identification, as shown in Figure 2c (I for original image captured by a bright‐field microscope, II for image treated by MTS, III for image processed by ImageJ) and 2d. This is because ImageJ only provides the number of directly recognized objects, while for the single PS_6_ counts, additional manual assistance is required. However, based on the training results, MTS can automatically split the aggregates into individual PS_6_ for counting and separately tracking their trajectories. The practical workflow of MTS is presented in Figure 2e. As time progresses, the trajectories of positive motors become gradually longer along the magnetic field direction. These results demonstrate that the self‐written MTS exhibits excellent identification and tracking accuracy, providing a powerful program to streamline the workflow of multi‐microparticle identification and trajectory tracking.

### 2.3 Feasibility and Analytical Performance of the Proposed AI‐dMIA

**FIGURE 3:**
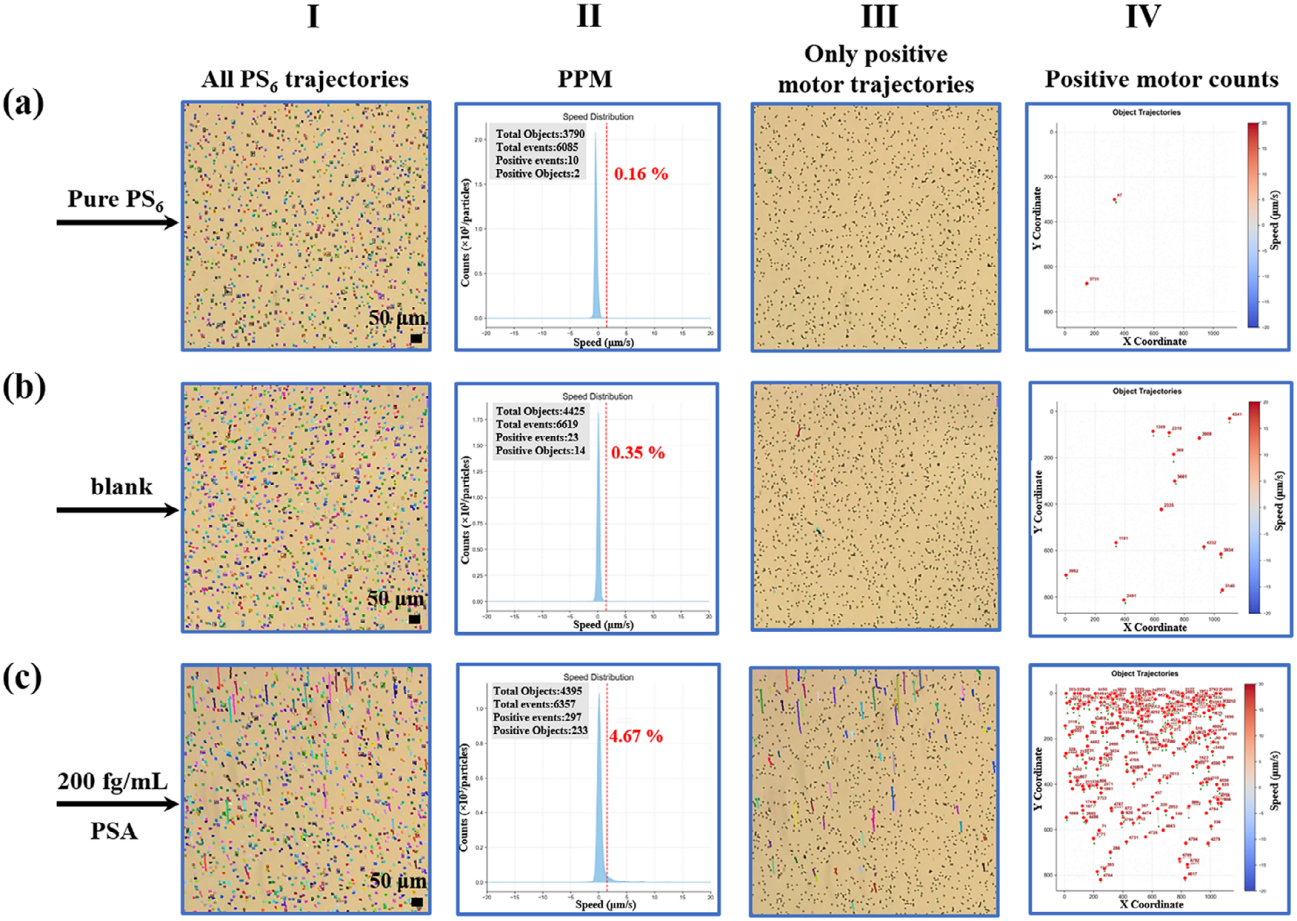
Feasibility test of the proposed AI‐dMIA by using PSA as the model target. The four columns represent the last frame image of all motor trajectories (recorded by a bright‐field microscope within 15 s after MTS analysis, Column I), percentage of positive motors (PPM, Column II), positive motor trajectories (identified and denoted in the corresponding last frame image, Column III), and positive motor counts (Column IV) of Pure PS_6_ (a), and the proposed sensing system with 0 (blank, b) and 200 fg/mL (c) PSA added. The threshold in Column II is set as the average motion speed of all pure PS_6_ plus 10 times the standard deviation (red line, 1.5 µm/s), to ensure almost all pure PS_6_ exhibit the Brownian motion and can be defined as negative. Scale bar: 50 µm.

Following the principles of dMIA and the MTS system demonstrated above, the feasibility and analytical performance of the AI‐dMIA are evaluated by adopting prostate‐specific antigen (PSA) as a model biomarker. To provide an ideal environment for the test, a liquid medium with proper density and viscosity is chosen to ensure that the PS_6_ can stably float on the liquid surface. This floating design greatly increases the motion speed of micromotors compared with that of the sunken micromotors on the solid substrate (Figure S1). Notably, the direction of the magnetic field (the magnetic field intensity simulation is shown in Figure S2) is fixed perpendicular to the lens. Hence, only the moving speed and trajectory of PS_6_ along the direction of the magnetic field are recorded for digital sensing purposes. First, a series of pure PS_6_ samples is examined to determine the threshold to differentiate positive/negative motors. As depicted in Figure 3a, the Brownian motion behavior of pure PS_6_ is analyzed by MTS, followed by selecting the average motion speed of all pure PS_6_ plus 10 times the standard deviation as the threshold (red line, Figure 3a). In this way, when the motion speed of a PS_6_ along the desired direction is greater than the set threshold, it will be identified as a positive motor (1). Conversely, the PS_6_ with motion speed lower than the threshold will be signed as negative (0). This setting can ensure that almost all the pure PS_6_ can be recognized as negative (the speed of positive and negative motors is shown in Figure S3). According to our test, when there are only PS_6_ and MNPs_50_ in the system to conduct the proposed AI‐dMIA without adding PSA (blank), only a negligible number of positive motors are detected, which may be attributed to the inevitably weak non‐specific adsorption between the antibody pair (Figure 3b). Nevertheless, when 200 fg/mL PSA is added into the AI‐dMIA system, significant positive motors showcase obvious motion behaviors and trajectories (Figure 3c), which can be clearly distinguished from the negative motors, evidencing that our proposed AI‐dMIA is feasible.

**FIGURE 4:**
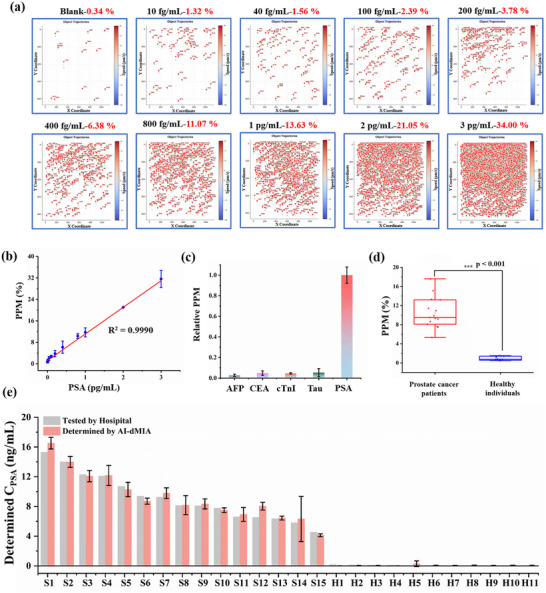
Analytical performance of the proposed AI‐dMIA. (a) The number of positive motors and PPM induced by different dosages of PSA, including 0, 10, 40, 100, 200, 400, 800, 1000, 2000, and 3000 fg/mL. (b) Linear relationship between the PPM and PSA concentrations. *n* = 3. (c) Investigating the specificity of the proposed AI‐dMIA by detecting different proteins, including AFP, CEA, cTnI, Tau, and PSA (400 fg/mL for all) by using the PSA‐specific antibodies, where the PPM induced by PSA is normalized to 1. *n* = 3. (d) Statistical analysis of the AI‐dMIA results for PSA detection (Student's *t*‐Test, ^***^ stands for *p* < 0.001). (e) PSA levels of 26 sera including 15 prostate cancer patients (S1∼15) and 11 healthy individuals (H1∼11), were determined by the hospital and the proposed AI‐dMIA. *n* = 3. Error bars represent the standard deviation from 3 parallel tests.

Under the optimal conditions (Figures S4–S6), the analytical performance of the AI‐dMIA is investigated by detecting a series of dilutions of PSA. As displayed in Figure 4a, the number of positive motors and PPM are progressively ascending with the concentrations of PSA rising from 10 fg/mL to 3 pg/mL (also see Movies S1–S3 for blank, 400 fg/mL, 2 pg/mL PSA, respectively). As low as 10 fg/mL PSA can be clearly distinguished from the blank control. An excellent linear curve is obtained by plotting the relationship between the PPM and PSA concentrations (0.01 to 3 pg/mL), the regression equation of which is *PPM* = 9.91 *C*_PSA_ (pg/mL) + 1.23, with a correlation coefficient R^2^ of 0.9990 (Figure 4b). The reproducibility of the proposed AI‐dMIA is evaluated by testing 400 fg/mL PSA on different days, which indicates an excellent performance (Figure S7). Moreover, the proposed AI‐dMIA is extended to quantify hepatocellular biomarker Alpha Fetoprotein (AFP) and Alzheimer's disease‐associated Protein Tau (Tau), for investigating its generality using the corresponding target‐specific antibodies. Similar to PSA analysis, the same threshold (1.5 µm/s) is set to distinguish positive and negative motors. As depicted in Figures S8 and S9, the number of positive motors and PPM are steadily rising with the ascending AFP and Tau concentrations (0.04–8 pg/mL for AFP, 0.01–6 pg/mL for Tau), and the lowest determined dosages of AFP and Tau are 40 and 10 fg/mL, respectively. These results reveal that the proposed AI‐dMIA demonstrates excellent generality. What is more, several potentially interfering proteins are selected to evaluate the specificity of the proposed AI‐dMIA with the PSA‐specific antibody pair. As displayed in Figure S10, only the sample with PSA added can exhibit distinct motion along the magnetic field direction. The PPMs derived from AFP, carcinoembryonic antigen (CEA), Tau, and cardiac troponin I (cTnI) are all negligible (Figure 4c), indicating our proposed AI‐dMIA is of high specificity. And the verification results of isotype control antibodies are shown in Figure S11. Furthermore, 26 serum samples, including 15 from prostate cancer patients and 11 from healthy individuals, were used to explore the practicability of the proposed assay. As the results of AI‐dMIA presented in Figure 4d,e, the PSA levels of cancer patients are obviously higher than those of healthy individuals, which are consistent with the hospital results, suggesting that the proposed strategy holds great promise for monitoring target proteins in real complex samples.

**FIGURE 5:**
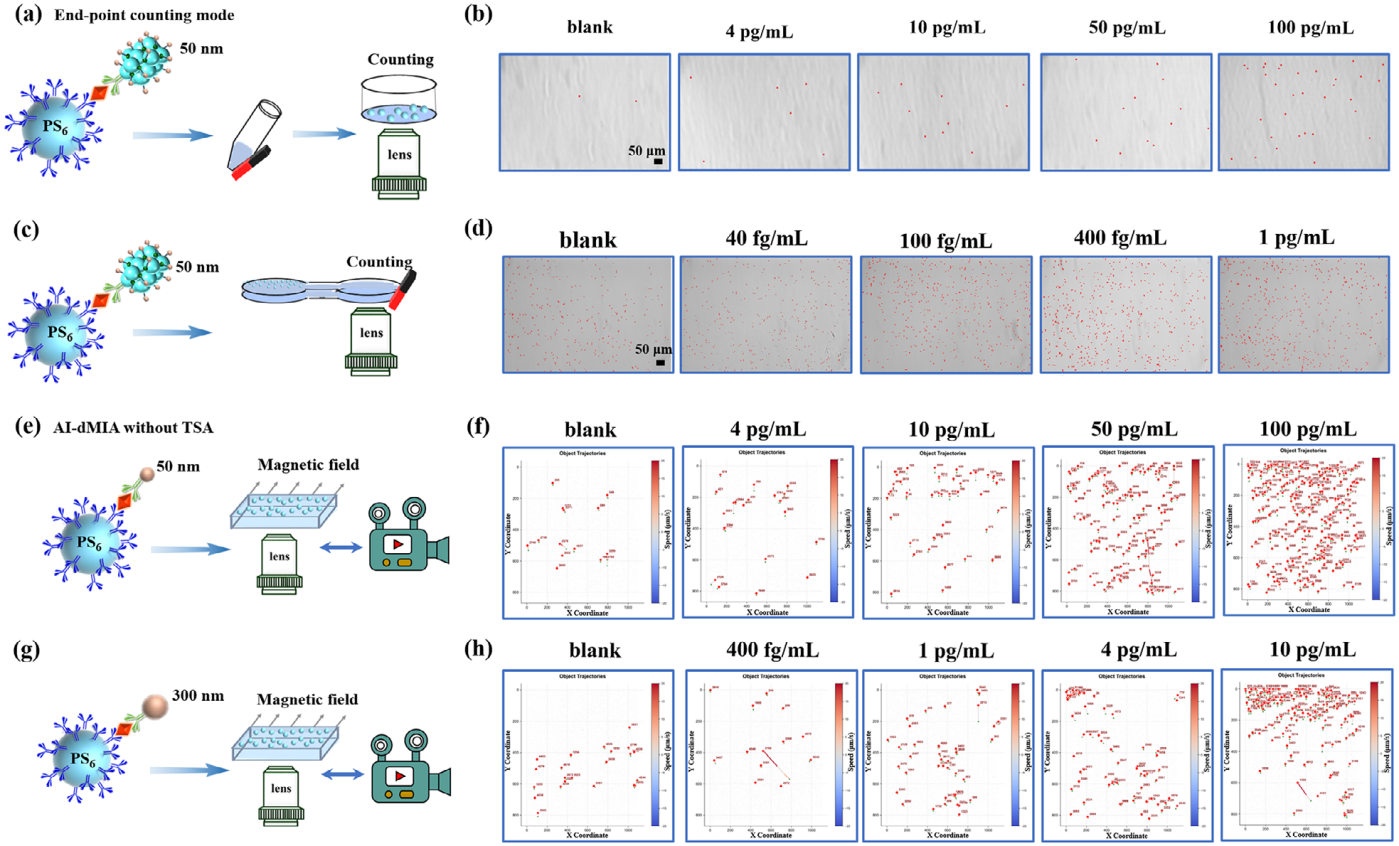
Verifying the advantages of the design. (a,b) The advantage of motion trajectory tracking over end ‐ point particle counting. (a) Schematic illustration of the end‐point detection mode by magnetic separation for PSA analysis. (b) The bright‐field images of PS_6_‐MNPs_50_ induced by different PSA dosages (0, 4, 10, 50, and 100 pg/mL), where the PS_6_‐MNPs_50_ are outlined by red circles. (c) The end ‐ point detection mode by using a double‐tank connected chip for PSA analysis. (d) The bright‐field images of PS_6_ in the right tank derived by different PSA concentrations (0, 40, 100, 400, and 1000 fg/mL), where the PS_6_ are labeled by red circles. (e‐h) The essential role of TSA in the AI‐dMIA. (e) TSA‐free one‐step immunoassay by using PS_6_ and Ab2‐MNP_50_ in the AI real‐time digital signaling manner. (f) The number of positive motors in (e) induced by different PSA dosages (0, 4, 10, 50, and 100 pg/mL). (g) TSA‐free one‐step immunoassay by using PS_6_ and Ab2‐MNP_300_ in the AI real‐time digital sensing manner. (h) The number of positive motors in (g) induced by different PSA concentrations (0, 0.4, 1, 4, and 10 pg/mL). Scale bar: 50 µm.

Currently, the existing digital immunoassays predominantly adopt the end‐point detection mode to count positive/negative entities in a static state after the whole process of signal generation/amplification reactions is completed. Different from the end‐point detection mode, this AI‐dMIA depends on a real‐time digital signaling manner and thus features unique superiorities. To demonstrate this, we attempt to analyze the same reaction system via end‐point magnetic separation. As depicted in Figure 5a, all PS_6_, including PS_6_‐MNPs_50_ and MNPs_50_‐free PS_6,_ are initially placed above the magnet. As such, the PS_6_‐MNPs_50_ will be attracted by the magnet. After 2 min, the supernatant is removed, and the precipitate is purified. Finally, the number of PS_6_‐MNPs_50_ in the precipitate is counted to reflect the dosages of PSA under the bright‐field microscope. As presented in Figure 5b, the number of PS_6_‐MNPs_50_ induced by 4 pg/mL PSA can just be differentiated from that of the blank control, which is far higher than the AI real‐time digital signaling manner (10 fg/mL). This can be attributed to the fact that at low abundance of PSA (or when following the Poisson distribution), only a tiny number of MNPs_50_ are conjugated on the PS_6_ surfaces by a single molecule immuno‐binding event. This results in insufficient magnetic force to guarantee the stable binding that is resistant to magnetic separation and washing. Only when there are a large number of immuno‐binding events on each PS_6_ can a sufficiently stable magnetic response and separation performance be achieved. To avoid the interference derived from magnetic separation and washing, we also design an alternative end ‐ point counting strategy to investigate the superiority of the AI real‐time digital sensing. As shown in Figure 5c, all the reacted PS_6_ are added into one of the tanks (illustrated as the left‐side one) of a double‐tank connected chip. Afterward, the PS_6_‐MNPs_50_ move into the other tank driven by a magnet. But a significant quantity of PS_6_ containing both PS_6_‐MNPs_50_ and MNPs_50_‐free PS_6_ will enter the other tank along the liquid flow, thus failing to reflect the actual target protein concentrations by simply counting the number of PS_6_‐MNPs_50_ (Figure 5d). Conversely, our AI‐dMIA avoids the magnetic separation and purification steps, which can precisely distinguish the PS_6_‐MNPs_50_ formed by even one single molecule immuno‐binding event from the pure PS_6_, indicating the AI real‐time digital signaling manner is superior to the end‐point signal readout routes.

In the proposed AI‐facilitated dMIA, TSA is the key to introducing sufficient MNPs_50_ to the single molecule binding site to form active micromotors. As depicted in Figure 5e, each target can only bind with one Ab2‐MNP_50_ by using the one‐step single‐molecule immunoreaction. Nevertheless, due to the small size and weak magnetism of MNPs_50_, at low target concentrations, the digital manner discrimination cannot be achieved. Only when the target concentration is higher than 10 pg/mL will there be obvious motion differences (Figure 5f). Then, we further employ MNPs_300_ (300 nm in diameter) with stronger magnetic responsiveness to carry out the one‐step single‐molecule immunoreaction. However, the analytical performance is still not as good as that of TSA‐MNPs_50_ (Figure 5g,h). Besides, a similar sensing performance can also be achieved by using MNPs_300_ instead of MNPs_50_ to perform the AI‐dMIA (Figure S12). Therefore, TSA plays a key character in guaranteeing the sensitivity of the proposed AI‐dMIA, which cannot be replaced by using large‐sized MNPs.

**FIGURE 6:**
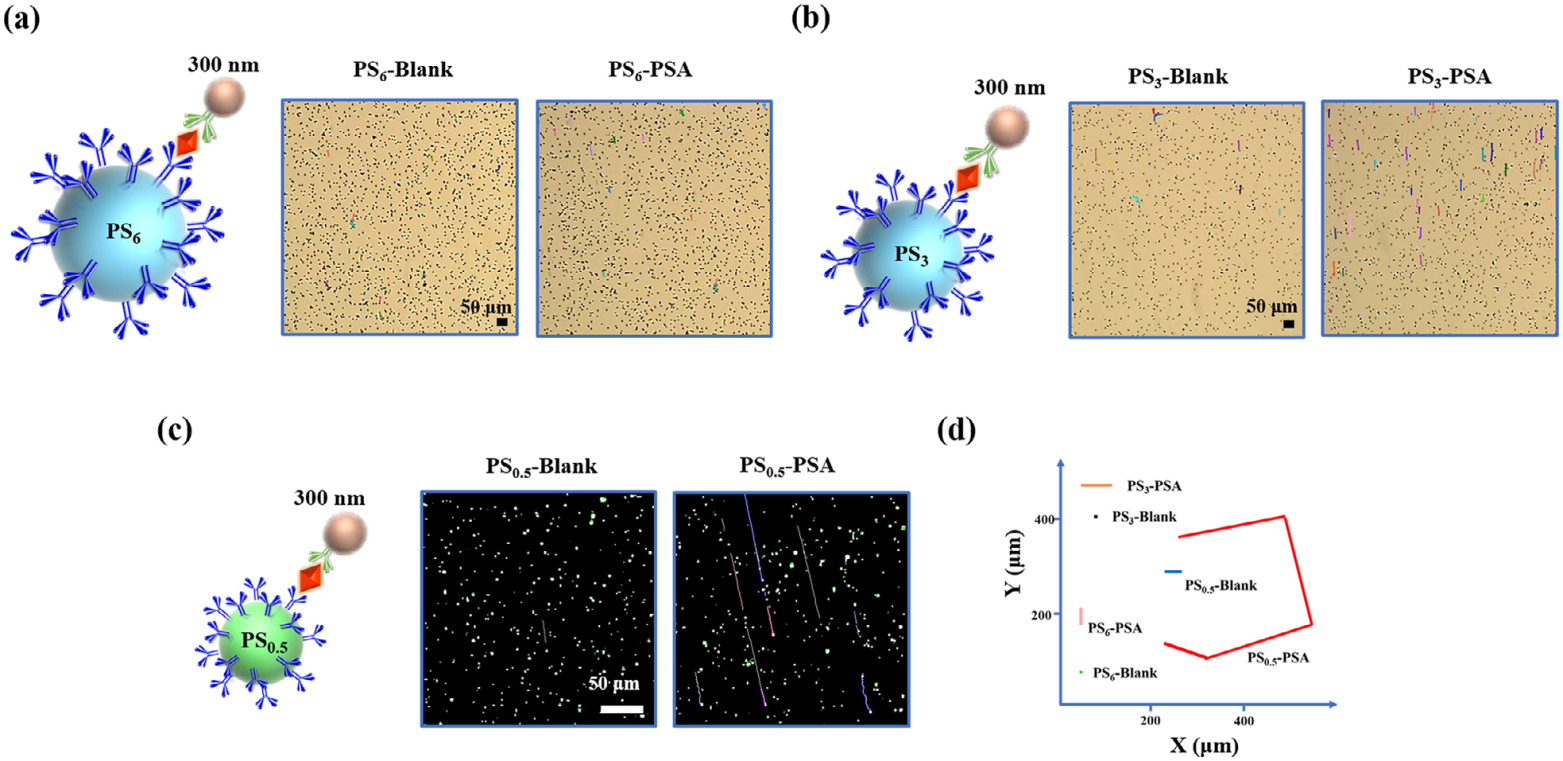
Motion trajectories of different‐sized PS. (a–c) One‐step sandwich immunoreaction and the trajectories induced by the blank control and PSA of PS_6_ and Ab2‐MNP_300_ (a), PS_3_ and Ab2‐MNP_300_ (b), and PS_0.5_ and Ab2‐MNP_300_ (c). (d) Comparison of the motion trajectories of PS_6_, PS_3_, and PS_0.5_ induced by the blank control and PSA. Scale bar: 50 µm.

The AI‐dMIA design in this study is demonstrated by using PS_6_ as the micromotor carrier. Actually, smaller PS‐based micromotors will exhibit much faster motion speed, which may be more easily and precisely discerned from negative pure PS. To verify this, we compared the motion trajectories of 3 µm PS (PS_3_) and 0.5 µm PS (PS_0.5_) by using the AI‐dMIA. It should be noted that PS_0.5_ is fluorescently labeled for the convenience of observation. Specifically, the PS_6_, PS_3_, and PS_0.5_ are tested via the one‐step sandwich immunoassay with Ab2‐MNP_300_, and the number of PSA molecules added in each group is the same as those of the corresponding PS. Their motion trajectories treated with the blank control and PSA are presented in Figure 6a–c, respectively. The trajectories of the motors in the blank control groups are similar and maintain the Brownian motion in place. Conversely, the target‐aroused trajectories of the differently sized micromotors show distinct differentiation. The trajectory of PS_0.5_ is the longest among the three sets of PS, whereas that of PS_6_ is the shortest. Moreover, the difference between the trajectories induced by blank and PSA is also the most prominent in the PS_0.5_ group, as displayed in Figure 6d. These results demonstrate that the smaller the diameter of the PS utilized, the more distinct the results obtained may be. Nonetheless, it is worth noting that the PS with a diameter of ∼500 nm is difficult to accurately monitor using a common bright‐field microscope. So, in most of the sensing scenarios, relatively large‐sized particles, such as PS_6_ and PS_3_ can fulfill the requirement. Particularly, by leveraging fluorescence‐labeled PS and fluorescence microscopes, fluorescent PS within a sub‐micrometer size can also be perfectly combined with our AI‐facilitated MTS, achieving accurate distinction between positive and negative motors, which presents great potential for point‐of‐care digital analysis.

## 3 Conclusion

In summary, we proposed an innovative AI‐dMIA, which employs the AI‐assisted MTS to track micromotor motion trajectories in real time for ultrasensitive quantification of target proteins in a digital way. In this design, a single‐molecule binding event on the confined target‐payload site of the micromotor can induce distinct motion trajectories, which are utilized as the positive signals, and the powerful MTS is capable of simultaneously tracking thousands of motion trajectories to distinguish positive and negative events in real time. Taking advantage of the unique design, the AI‐dMIA not only exhibits excellent sensing performance, which is similar to the Simoa in the aspects of sensitivity, time cost, and digital dynamic range, but also avoids the requirement of complicated operation, special reagents, as well as high‐end equipment that are essential to traditional end‐point digital bioassays.

It is also worth noting that the AI‐dMIA has some shortcomings at the current stage. For example, it relies on the bright‐field microscope to image and record the micromotor motion trajectories. Despite convenience and the prevalence in common laboratories, it has limitations in throughput and small‐sized particles, such as sub‐micrometer ones. Moreover, the suspension system used in this method requires compatibility with the material and size of the microspheres, and the calibration of thresholds in different experimental environments is not avoidable. Therefore, we hope to develop standard devices to improve the throughput of the AI‐dMIA and further combine it with fluorescence microscopes for a one‐step binding‐based simple digital assay. If all these issues can be well addressed, this proposed AI‐dMIA may pioneer a new direction for devising micromotor digital bioassays to make an advancement for trace biomarker analysis.

## 4 Experimental Section

### 4.1 Materials and Reagents

Carboxyl‐modified polystyrene microspheres (6 µm, PS_6_; 3 µm, PS_3_; 500 nm, PS_0.5_; ρ ≈ 1.06 g/cm^3^) were purchased from Bangs Laboratories, Inc. (USA). Streptavidin‐labeled magnetic nanoparticles (50 nm, MNPs_50_) were acquired from Ocean Nano Tech. (USA), and SA‐MNPs (300 nm, MNPs_300_) were obtained from Ademtech (France). 1‐(3‐Dimethylaminopropyl)‐3‐ethylcarbodiimide (EDC) and N‐Hydroxysulfosuccinimide sodium salt (sulfo‐NHS) were obtained from Sigma–Aldrich (USA). Prostate‐specific antigen (PSA), alpha fetoprotein (AFP), and corresponding antibody pairs, including monoclonal capture antibody (Ab1) and biotin‐modified monoclonal detection antibody (bio‐Ab2), carcinoembryonic antigen (CEA), as well as cardiac troponin I (cTnI), were obtained from Shanghai Linc‐Bio Science Co., Ltd (China). Alzheimer's disease‐associated Protein Tau (Tau) and the corresponding antibody pair were purchased from Biolegend, Inc. (USA). Streptavidin‐polyHRP (SA‐polyHRP) was obtained from Thermo Fisher (USA). A biotin‐tyramide signal amplification kit was acquired from Biodragon (China). Cesium chloride (CsCl) was purchased from J&K Scientific (China). Methylcellulose was acquired from Shanghai Aladdin Biochemical Technology Co., Ltd. (China).

### 4.2 Preparation of Capture Antibody‐Conjugated PS_6_ (Ab1‐PS_6_)

First, a strong vortex was conducted to ensure that the PS_6_ (6 µm) was well dispersed. 2.5 µL of PS_6_ was taken out and dispersed into MEST buffer (25 mm, pH 5.0, 0.05% Tween‐20), and then washed twice. Afterward, the carboxyl groups on PS_6_ surfaces were activated in MES buffer (25 µL) containing 12.5 µL of EDC (50 mg/mL) and 12.5 µL of sulfo‐NHS (50 mg/mL). After 30 min, the activated PS_6_ were washed once using MEST, and then incubated with 1 µg of capture antibody (Ab1) for 2 h to form Ab1‐labeled PS_6_ (Ab1‐PS_6_). Subsequently, 1% BSA and 50 mm Tris (pH 8.0) were added to block the active sites on PS_6_ surfaces. After 0.5 h, the Ab1‐PS_6_ were washed three times and stored in 25 µL of 1 × PBST‐BSA (1 × PBS, 0.1% Tween‐20, and 1% BSA) for future use.

### 4.3 Typical Procedures of the Proposed AI‐dMIA

PSA was selected as a model analyte to describe the typical procedures of the AI‐dMIA. The PSA‐specific capture antibody‐modified PS_6_ (Ab1‐PS_6_, 7.8 × 10^4^ particles) were used in the sandwich immunoreaction with 0.2 ng PSA‐specific bio‐Ab2 and different PSA dosages for 1 h under vibration at room temperature. Afterward, Ab1‐PS_6_ were incubated with 0.5 ng SA‐polyHRP for 30 min at room temperature. After washing, such Ab1‐PS_6_ were dispersed into the TSA buffer, including 1 × tyramine dilution solution, and 2 ng biotin‐labeled tyramine to execute the deposition of biotin‐tyramine near polyHRP under the catalysis of H_2_O_2_. Subsequently, SA‐MNPs_50_ (2 × 10^7^ particles) were introduced to be immobilized on the PS_6_ surface to form PS_6_‐MNPs_50_ magnetic micromotors. Conversely, target‐free PS_6_ will not be bound with MNPs_50_ (MNPs_50_‐free PS_6_). Then, the PS_6_‐MNPs_50_ and MNPs_50_‐free PS_6_ were isolated and then dispersed into 4 µL of the testing liquid medium, including 25% CsCl (ρ ≈ 1.15 g/cm^3^) [33] supplemented with 0.01% methylcellulose and 0.05% Tween‐20. The above mixture was added into a square‐well chip (4 mm × 4 mm), which was placed in a fixed magnetic field when all PS_6_ were in the floating state. After 15 s magnetic response, the motor motion video was recorded by a Laica bright‐field microscope equipped with an MC170 camera and a 5 × objective. Finally, the motion video was analyzed by the MTS to output a new trajectory video and PPM to reflect the target protein level. As for PS_0.5_, the testing liquid medium contained 25% CsCl supplemented with 0.25% methylcellulose and 0.05% Tween‐20. The PS_0.5_ were imaged by an Olympus IX‐71 microscope equipped with a xenon lamp with a U‐FBVW mirror unit. The motion video was recorded using the same microscope equipped with a DP73 camera (Olympus) and a 10 × objective. Afterward, the motion videos were enhanced by using Self‐Calibrated Illumination (SCI) [34] before being analyzed by MTS.

### 4.4 Determining PSA Levels in Serum Samples by the Proposed AI‐dMIA

26 serum samples, including 15 from prostate cancer patients and 11 from healthy individuals (provided by the First Affiliated Hospital of Zhengzhou University (KY‐2021‐0358)) were employed to investigate the practicability of the AI‐dMIA. The samples were diluted 10 000 times before the measurement.

### 4.5 Procedures of the One‐Step Sandwich Immunoassay by Using PS_6_ and Ab2‐MNP_300_

Ab1‐PS_6_ were incubated with Ab2‐MNPs_300_ and PSA (the number of molecules was the same as that of the PS_6_) to conduct one‐step sandwich immunoassays for forming magnetic micromotors under vibration at ambient temperature for 2 h. Then, the mixture was placed into the testing liquid to perform the magnetic‐driven motor motion in the fixed magnetic field under a bright‐field microscope. Finally, the recorded motion videos were analyzed by MTS for PSA detection. The procedures of a one‐step sandwich immunoassay by using PS_3_ (or PS_0.5_) and Ab2‐MNP_300_ are similar to those of PS_6_. Additionally, the motion trajectories of PS_0.5_ were recorded by using a common fluorescence microscope with a xenon lamp.

### 4.6 Procedures of the AI‐dMIA by Using PS_6_ and MNPs_300_

The Ab1‐PS_6_ were incubated with PSA and bio‐Ab2 to perform the sandwich immuno‐reaction, followed by executing TSA to deposit numerous bio‐tyramine molecules, which was the same as the typical procedures of the AI‐dMIA. Then, MNPs_300_ were introduced into the above system to be immobilized on PS_6_ surfaces to form PS_6_‐MNPs_300_ magnetic micromotors. Finally, the PS_6_ were placed into the same testing liquid to drive the motor motion in the magnetic field under a bright‐field microscope. The recorded motion videos were directly processed by the MTS for PSA analysis.

### 4.7 Testing Solution

As for PS_6_ and PS_3_, the testing liquid consists of 25% CsCl (ρ ≈ 1.15 g/cm^3^) supplemented with 0.01% methylcellulose and 0.05% Tween‐20. As for PS_0.5_, the testing liquid consists of 25% CsCl, 0.25% methylcellulose, and 0.05% Tween‐20.

### 4.8 Statistical Analysis

The motion videos were analyzed using Python. Statistical analyses were performed using Microsoft Office Excel. Data were expressed as mean ± SD, and sample size (*n*) for each statistical analysis was represented in the corresponding figure legends. All statistical analyses were performed with Student's *t*‐Test for comparisons between differentially expressed proteins in prostate cancer patients and healthy individuals. Results were considered to show significant differences if the *p*‐value was < 0.001.

## Author Contributions

J.S. performed in data acquisition and analysis and wrote the original manuscript. Z.Y. and H.T. performed in developing AI‐facilitated MTS, as well as writing and revising the corresponding parts. W.F. and Y.S. performed in data analysis. W.R. performed in supervision, reviewing, editing, and funding acquisition. C.L. performed in design and guidance, writing revision and editing, providing resources, and funding acquisition. All authors provided feedback and edits.

## Conflicts of Interest

The authors declare no conflicts of interest.

## Supporting information




**Supporting File 1**: advs74545‐sup‐0001‐SuppMat.docx.

**Supporting File 2**: advs74545‐sup‐0002‐MovieS1.mp4.

**Supporting File 3**: advs74545‐sup‐0003‐MovieS2.mp4.

**Supporting File 4**: advs74545‐sup‐0004‐MovieS3.mp4.

## Data Availability

The data that support the findings of this study are available in the supplementary material of this article.
