# Supplementary material for: Real‐Time Digital Micromotor Tracking‐Enabled Ultrasensitive Immunoassay
Source: Adv Sci (Weinh). 2026 Feb 21;13(25):e21243. doi: 10.1002/advs.202521243 (PMC13137832; doi:10.1002/advs.202521243)
Supplement: Supplementary file 1 — Supporting File 1: advs74545‐sup‐0001‐SuppMat.docx. [file ADVS-13-e21243-s004.docx]

**Supporting Information**

**Real-Time Digital Micromotor Tracking-enabled Ultrasensitive Immunoassay**

*Jingjing Shi^a^, Zuhua Yu^b^, Hui Tian^b*^, Wenjiao Fan^a^, Yuanyuan Sun^c^, Wei Ren^a*^, Chenghui Liu^a*^*

J. Shi, W. Fan, W. Ren, C. Liu

School of Chemistry & Chemical Engineering

Shaanxi Normal University

Xi’an 710119, P. R. China

Email: wei.ren@snnu.edu.cn; [liuch@snnu.edu.cn](mailto:liuch@snnu.edu.cn)

Z. Yu, H. Tian

School of Cyber Science and Engineering

Zhengzhou University

Zhengzhou 450001, P. R. China

Email: [tianhui@zzu.edu.cn](mailto:tianhui@zzu.edu.cn)

Y. Sun

Department of Translational Medicine Center

The First Affiliated Hospital of Zhengzhou University

Zhengzhou 450052, P. R. China

**List of contents**

1. 10-fold cross-validation results
2. Evaluating the tracking performance of MTS
3. Magnetic micromotor motion in the floating and sinking states
4. Magnetic field intensity simulation
5. Comparison of the motion speeds of positive and negative motors
6. Optimization of experimental conditions for PSA analysis with the proposed AI-dMIA
7. Reproducibility of the AI-dMIA
8. Generality test of the AI-dMIA with AFP analysis
9. Generality test of the AI-dMIA with Tau analysis
10. Evaluating the specificity of the proposed AI-dMIA
11. Verification of the specific recognition of PSA by using isotype control antibodies
12. Detection results of the proposed AI-dMIA by using PS_6_ and MNPs_300_ for PSA analysis
13. **10-fold cross-validation results**

A total of 1210 images are divided into 10 subsets. In 10 independent iterations, one subset (10%) is sequentially selected as a completely independent test set, ensuring that each image is tested exactly once. The remaining 90% of the data is further partitioned into a training set (70%) and a validation set (20%), ensuring that the data division ratio in each round is consistent with the original study. As shown in Table S1, the Average Precision of the model on the independent test set is as high as 97.76%, with a standard deviation of only 2.46%. Such a minimal fluctuation indicates that the model performance is highly consistent and robust across different data partitions. The comprehensive results suggest that the current dataset size has not led to overfitting, and the model has strong generalization ability.

**Table S1.** 10-fold cross-validation results.

| **Metric** | **Mean** | **Standard Deviation** |
| --- | --- | --- |
| Precision | 98.07% | $\pm$0.76% |
| Recall | 95.69% | $\pm$3.78% |
| Average Precision | 97.76% | $\pm$2.46% |

1. **Evaluating the tracking performance of MTS**

Tracking metrics and the potential risk of overfitting due to the small sample size are crucial for evaluating the reliability of the method. The standard multiple objects tracking metrics are calculated on the test set using fully annotated video sequences. As shown in Table S2, the method achieved an MOTA of 92.72% and MOTP of 51.00%. For the evaluation of the identity stability, the Identification F1-Score (IDF1) rate was up to 93.21% with a Most Tracked (MT) rate of 85.64%. These results indicate that MTS possesses high multi-object tracking accuracy and robust identity preservation capabilities for the majority of micromotor trajectories at long durations.

**Table S2.** Comprehensive tracking performance.

| **Metric Category** | **Metric** | **Value** |
| --- | --- | --- |
| Tracking Accuracy | MOTA | 92.72% |
| Tracking Precision | MOTP | 51.00% |
| Identity Stability | IDF1 | 93.21% |
| Trajectory Quality | MT | 85.64% |
|  | ML | 1.10% |

1. **Magnetic micromotor motion in the floating and sinking states**

Considering that the moving speed of micromotors greatly varies in different media, we compare the micromotor motion in the floating state and when they are sunken on a solid substrate by conducting the proposed AI-dMIA. As shown in Figure S1, when all the PS_6_ are placed into the PBST buffer and sunken on a solid substrate (the bottom of the square well), little micromotor motion is detected in the presence of 400 fg/mL PSA, which is difficult to distinguish from the blank control (Figure S1a). Conversely, when all the PS_6_ are placed into the high-density testing liquid (25% CsCl (ρ ≈ 1.15 g/cm^3^), 0.01% methylcellulose, and 0.05% Tween-20) that can ensure they remain floating, numerous micromotors exhibit obvious motion trajectories in the presence of 400 fg/mL PSA (Figure S1b), which is obviously higher than those of the blank control. These results indicate that the movement resistance of micromotors is greatly reduced in the floating state, thus increasing the motion speed for the clear separation of positive and negative motors.

**
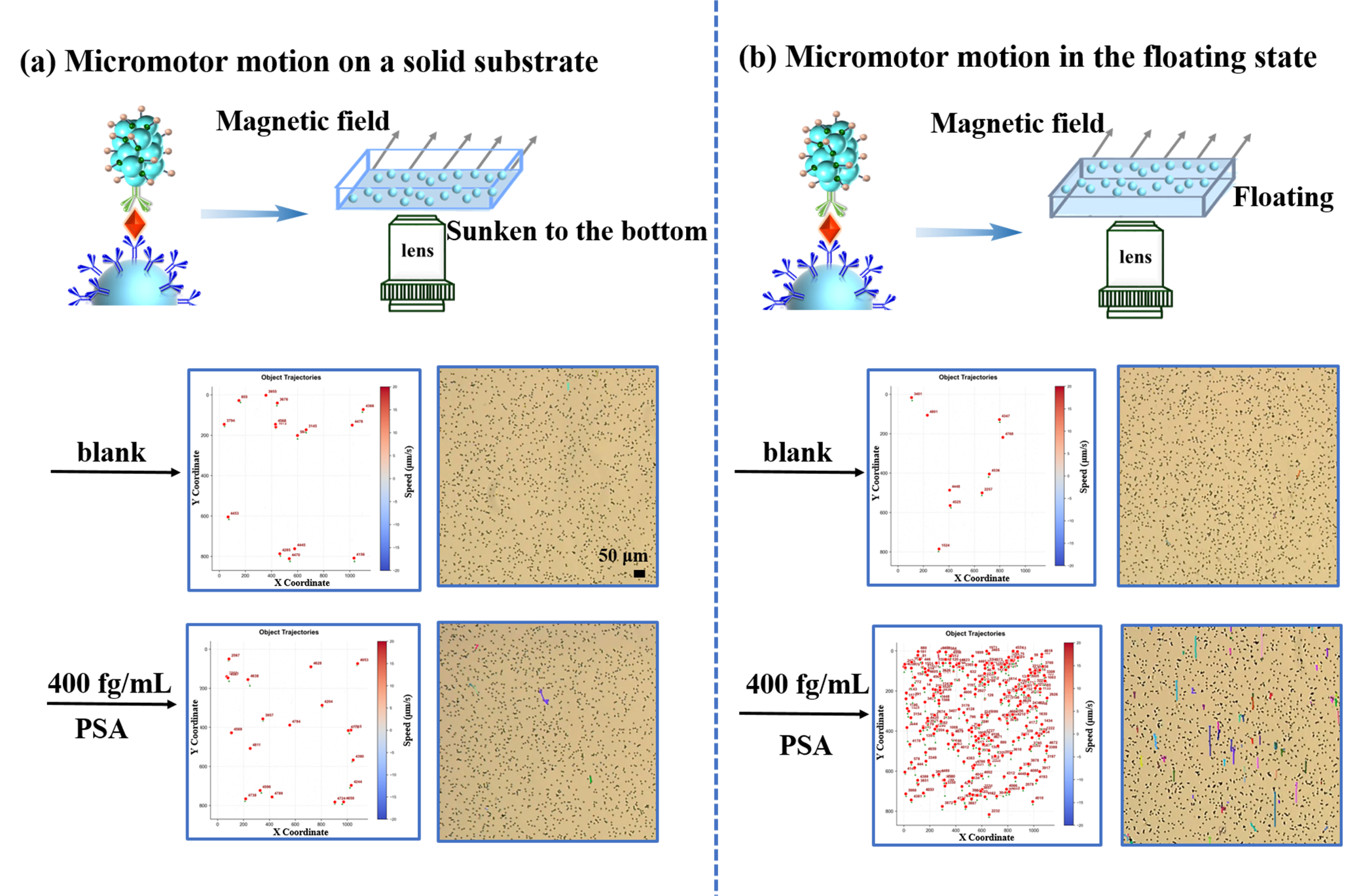
**

**Figure S1.** (a) Cartoon illustration of observing micromotor motion on a solid substrate (sunken to the bottom of the square well), and the positive counts and motion trajectories induced by 0 (blank) and 400 fg/mL PSA. The threshold is set as the average speed of pure PS_6_ plus ten times the standard deviation on the solid substrate. (b) Cartoon illustration of observing micromotor motion in the floating state, and positive counts and motion trajectories induced by 0 (blank) and 400 fg/mL PSA. The threshold is set as the average speed of pure PS_6_ plus 10 times the standard deviation in the floating state. Scale bar: 50 μm.

1. **Magnetic field intensity simulation**

As shown in Figure S2, the material of the magnet used in this work is NdFeB-N35, and the length, width, and height of the rectangular magnet are respectively 60 mm×10 mm×5 mm. The magnetic field strength at the edge of the magnet is 0.23 T, while in the middle it is 0.11 T.

**
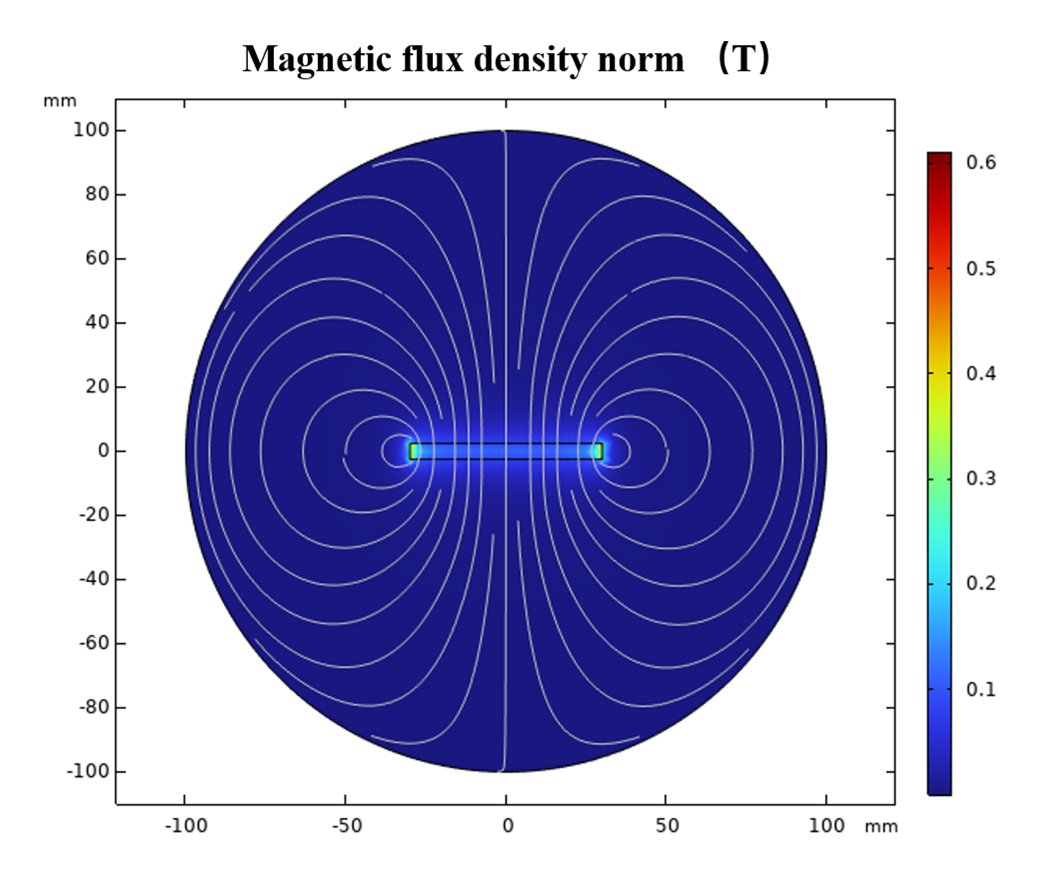
**

**Figure S2.** Magnetic flux density of the magnetic field used in this work.

1. **Comparison of the motion speeds of positive and negative motors**

**
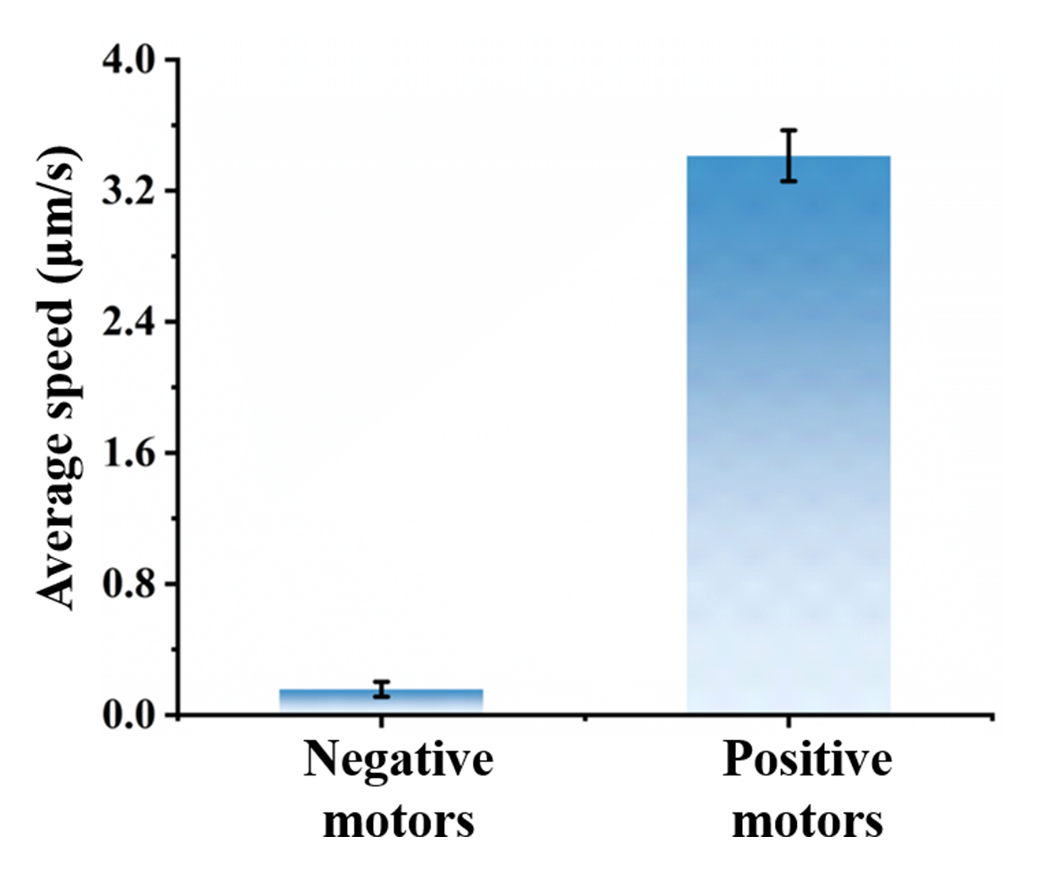
**

**Figure S3.** Comparison of the average speeds of positive and negative motors. n = 3. Error bars represent the standard deviation from 3 parallel tests.

1. **Optimization of experimental conditions for PSA analysis with the proposed AI-dMIA**

To acquire the best analytical performance of this proposed AI-dMIA for PSA analysis, the amounts of bio-tyramine, bio-Ab2, and MNPs_50_ were optimized, respectively.

First, different amounts of bio-tyramine (0.1, 0.5, 1, 2, and 5 ng) were subjected to TSA to be deposited onto the target-payload site after PSA-specific sandwich immunoreactions, further binding with MNPs_50_ to form PS_6_-MNPs_50_ magnetic motors. As illustrated in Figure S4a and S4b, both the number of positive motors and PPM induced by the blank control and PSA exhibit a gradual increase in the bio-tyramine dosage range of 0.5~5 ng. Notably, there is a significant increase in PPM in the blank control at 5 ng due to the non-specific random deposition of bio-tyramine molecules. Meanwhile, the ΔPPM (defined as PPM_PSA_-PPM_blank_) is evaluated and shown in Figure S4c. It can be observed that the ΔPPM increases as the bio-tyramine ascends from 0.1 to 2 ng, but decreases when the bio-tyramine dosages further rise from 2 to 5 ng. Therefore, the amount of the bio-tyramine is optimized as 2 ng.

**
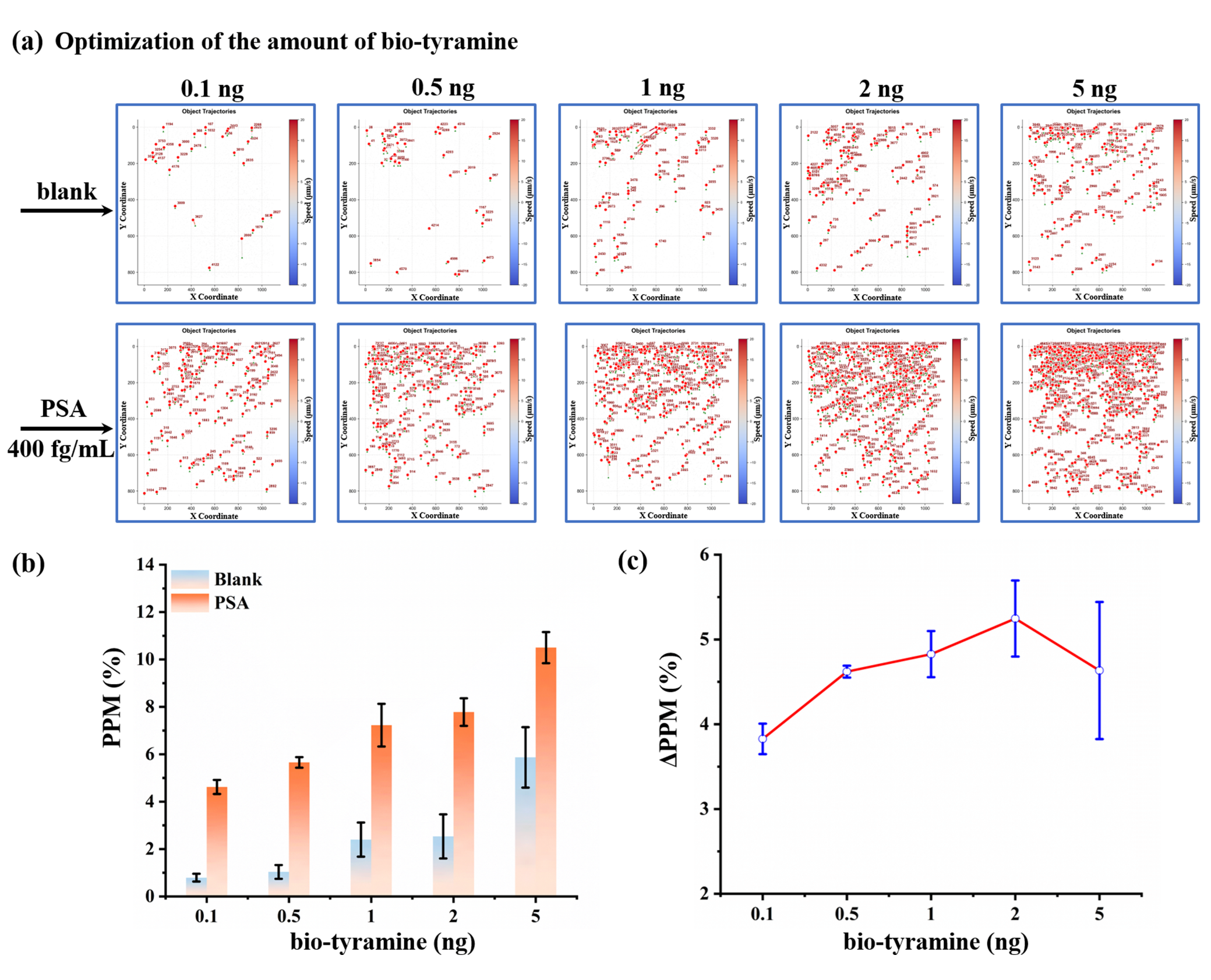
**

**Figure S4.** Optimization of the amount of bio-tyramine. (a) The number of positive motors produced by 0 (blank) and 400 fg/mL PSA under different amounts of bio-tyramine (0.1, 0.5, 1, 2, and 5 ng). (b) PPM produced by the 0 (blank) and 400 fg/mL PSA. n = 3. (c) The corresponding ΔPPM (defined as PPM_PSA_-PPM_blank_). n = 3. The amount of bio-Ab2 and MNPs_50_ is 0.5 ng and 2.5×10^7^ particles, respectively. Error bars represent the standard deviation from 3 parallel tests.

Under the optimal amount of the bio-tyramine, the dosages of bio-Ab2 were further optimized using five gradients: 0.05, 0.1, 0.2, 0.5, and 1 ng. As depicted in Figure S5a and S5b, both the number of positive motors and PPM aroused by the blank control and PSA are progressively ascending as the amount of bio-Ab2 increases from 0.05 to 1 ng, and of the two parameters of the blank control rise sharply in the range of 0.5 and 1 ng, which can be attributed to the non-specific absorption between the antibody pair. Moreover, the ΔPPM was assessed, and the results are presented in Figure S5c. The ΔPPM ascends in the range of 0.05 to 0.2 ng bio-Ab2, but declines when the amount of bio-Ab2 continues to rise from 0.2 to 1 ng. Therefore, 0.2 ng of bio-Ab2 was selected for the AI-dMIA.


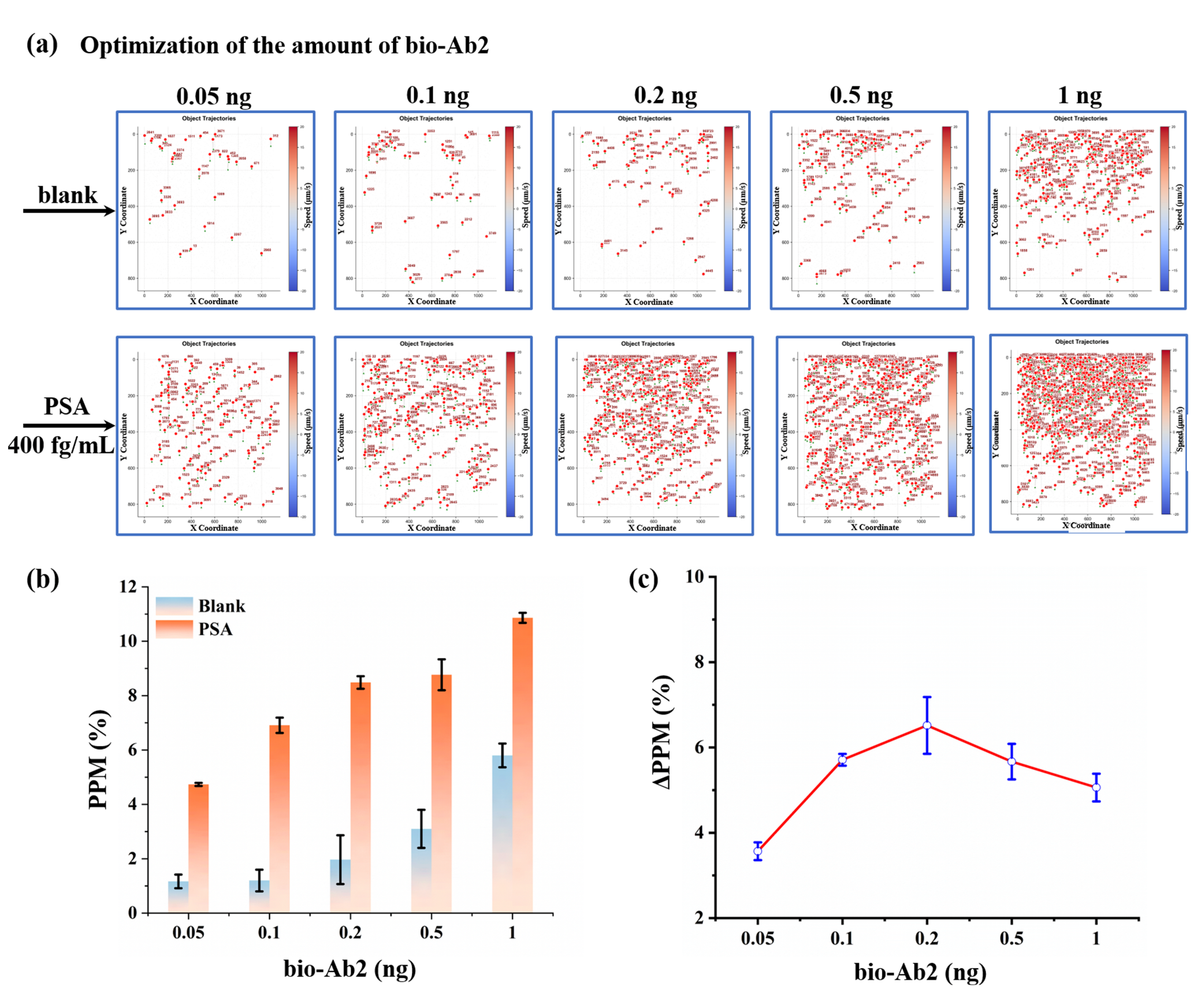


**Figure S5.** Optimization of the amount of bio-Ab2. (a) The number of positive motors produced by 0 (blank) and 400 fg/mL PSA with different amounts of bio-Ab2 (0.05, 0.1, 0.2, 0.5, and 1 ng). (b) PPM produced by the 0 (blank) and 400 fg/mL PSA. n = 3. (c) The corresponding ΔPPM. n = 3. The amount of MNPs_50_ is 2.5×10^7^ particles. Error bars represent the standard deviation from 3 parallel tests.

Under the above conditions, the amount of MNPs_50_ was optimized by conducting the proposed AI-dMIA for PSA analysis with 1×10^7^, 1.5×10^7^, 2×10^7^, 2.5×10^7^, and 5×10^7^ particles of MNPs_50,_ respectively. As displayed in Figure S6a and S6b, the number of positive motors and PPM triggered by the blank control and PSA show a gradual upward trend as the amount of MNPs_50_ increases from 1×10^7^ to 5×10^7^ particles. Notably, the positive motor counts of the blank control show a significant elevation at 2.5×10^7^ and 5×10^7^ particles. Additionally, the ΔPPM was investigated and displayed in Figure S6c. One can see that the ΔPPM increases from 1×10^7^ to 2×10^7^ particles and then decreases from 2×10^7^ to 5×10^7^ particles. Hence, the optimal amount of MNPs_50_ is 2×10^7^ particles.


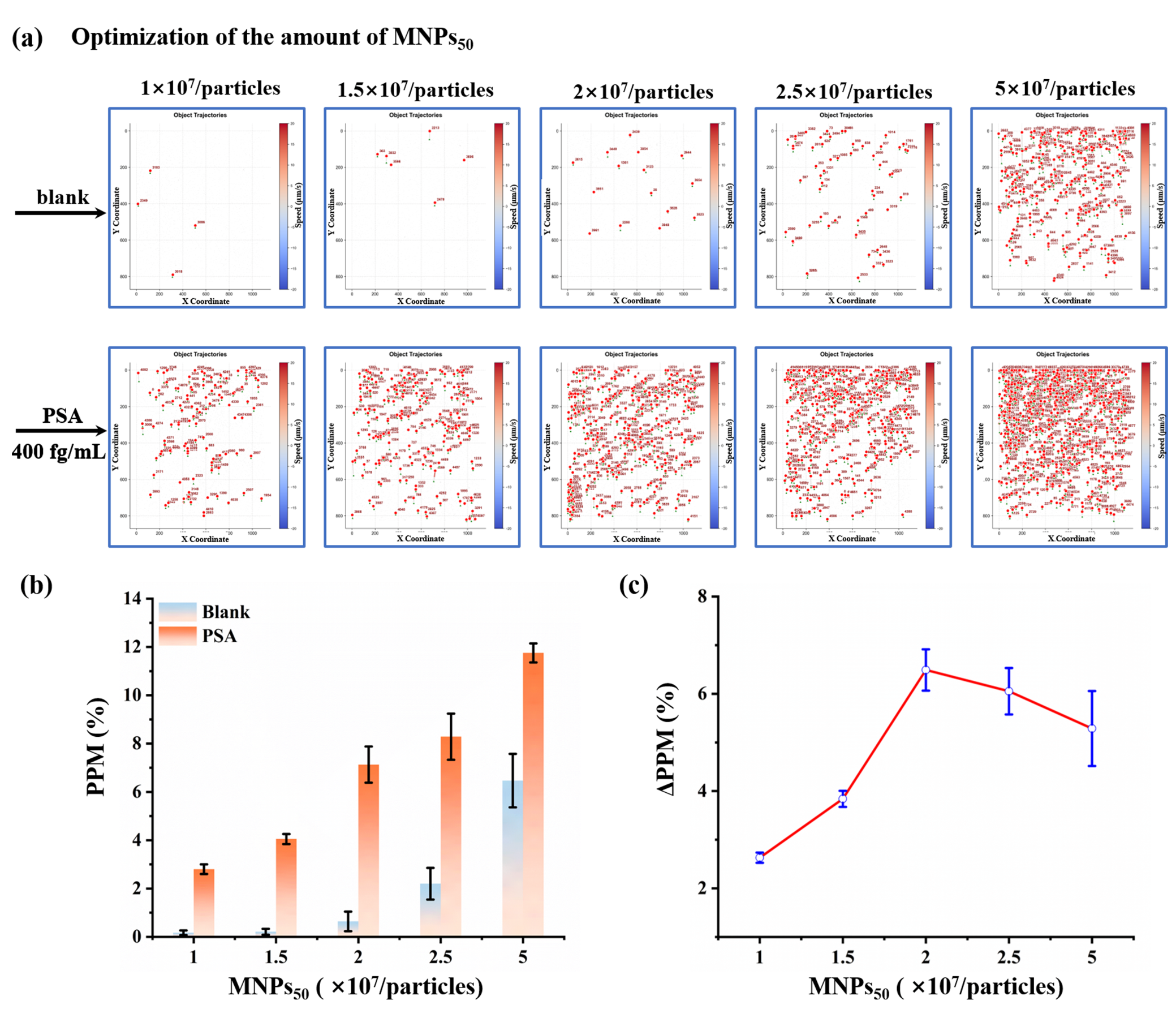


**Figure S6.** Optimization of the amount of MNPs_50_. (a) The number of positive motors produced by 0 (blank) and 400 fg/mL PSA with different amounts of MNPs_50_ (1×10^7^, 1.5×10^7^, 2×10^7^, 2.5×10^7^, and 5×10^7^ particles). (b) PPM produced by the 0 (blank) and 400 fg/mL PSA. n = 3. (c) The corresponding ΔPPM. n = 3. Error bars represent the standard deviation from 3 parallel tests.

The finally obtained optimal experimental conditions for PSA analysis by the AI-dMIA are 2 ng bio-tyramine, 0.2 ng bio-Ab2, and 2×10^7^ particles MNPs_50_, respectively. Moreover, these experimental conditions are also applied in AFP and Tau analysis by use of the proposed AI-dMIA.

1. **Reproducibility of the AI-dMIA**

**
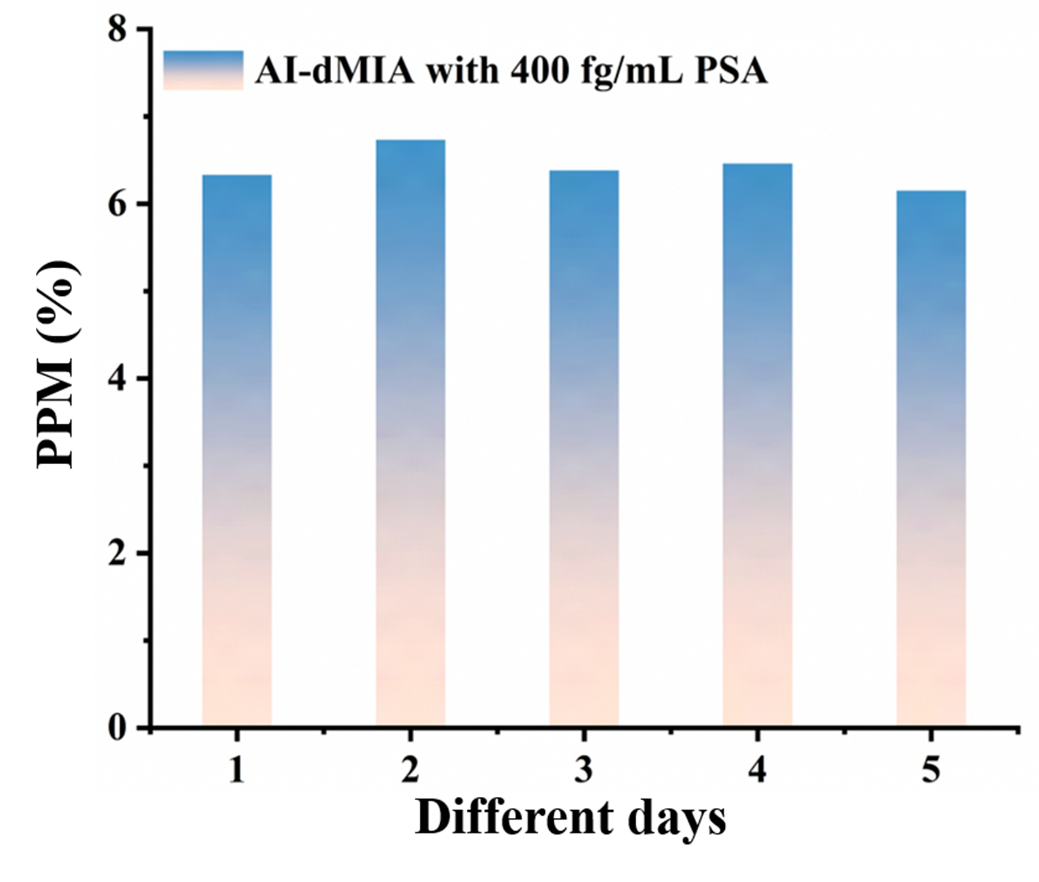
**

**Figure S7.** Reproducibility of the AI-dMIA when testing 400 fg/mL PSA from 5 different days.

1. **Generality test of the AI-dMIA with AFP analysis**

The generality of the proposed AI-dMIA is investigated by analyzing AFP. As depicted in Figure S8a, the number of positive motors and PPM is steadily rising with the ascending AFP concentrations in the range of 0.04~8 pg/mL. The lowest determined dosage of AFP is 40 fg/mL, and a favorable standard curve is obtained in AFP detection. The corresponding linear equation is *PPM* = 3.93 *C*_AFP_ (pg/mL) + 1.30 (R^2^ of 0.9913, Figure S8b), which reveals that the proposed AI-dMIA demonstrates excellent generality.


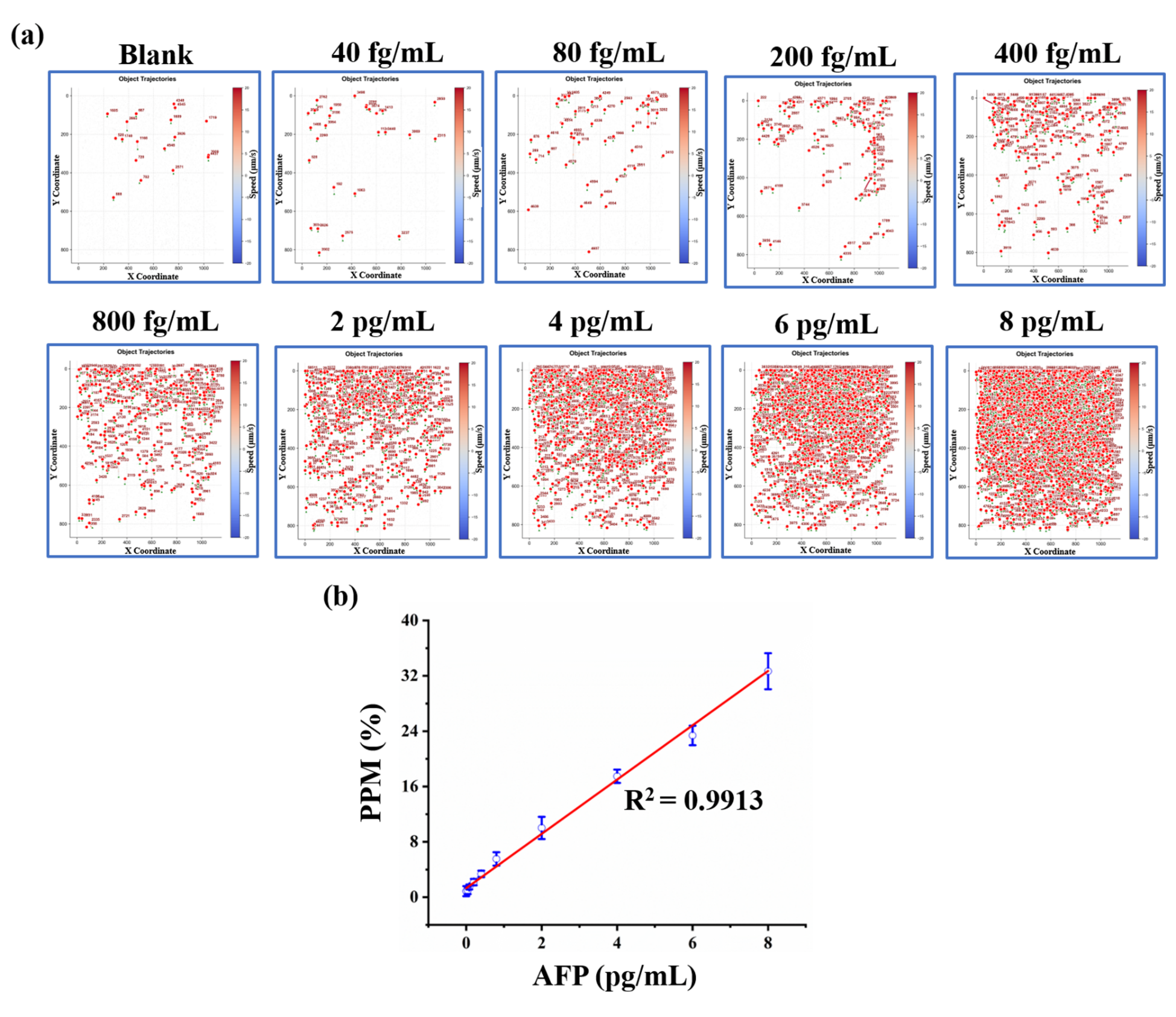


**Figure S8.** Investigating the generality of the AI-dMIA for AFP analysis. (a) The number of positive motors induced by different AFP concentrations (0, 0.04, 0.08, 0.2, 0.4, 0.8, 2, 4, 6, and 8 pg/mL). (b) Linear relationship between the PPM and AFP dosages (0~8 pg/mL). n = 3. Error bars represent the standard deviation from 3 parallel tests.

1. **Generality test of the AI-dMIA with Tau analysis**

The generality of the proposed AI-dMIA is also investigated by detecting Tau. As presented in Figure S9a, the number of positive motors and PPM are gradually increasing with the ascending Tau concentrations (0.01~6 pg/mL). As low as 10 fg/mL Tau can be distinguished from the blank control, and a good linear curve is obtained in Tau analysis. The corresponding linear equation is *PPM* = 6.06 *C*_Tau_ (pg/mL) + 1.03 (R^2^ of 0.9900, Figure S9b), which indicates that the proposed AI-dMIA demonstrates good generality.

**
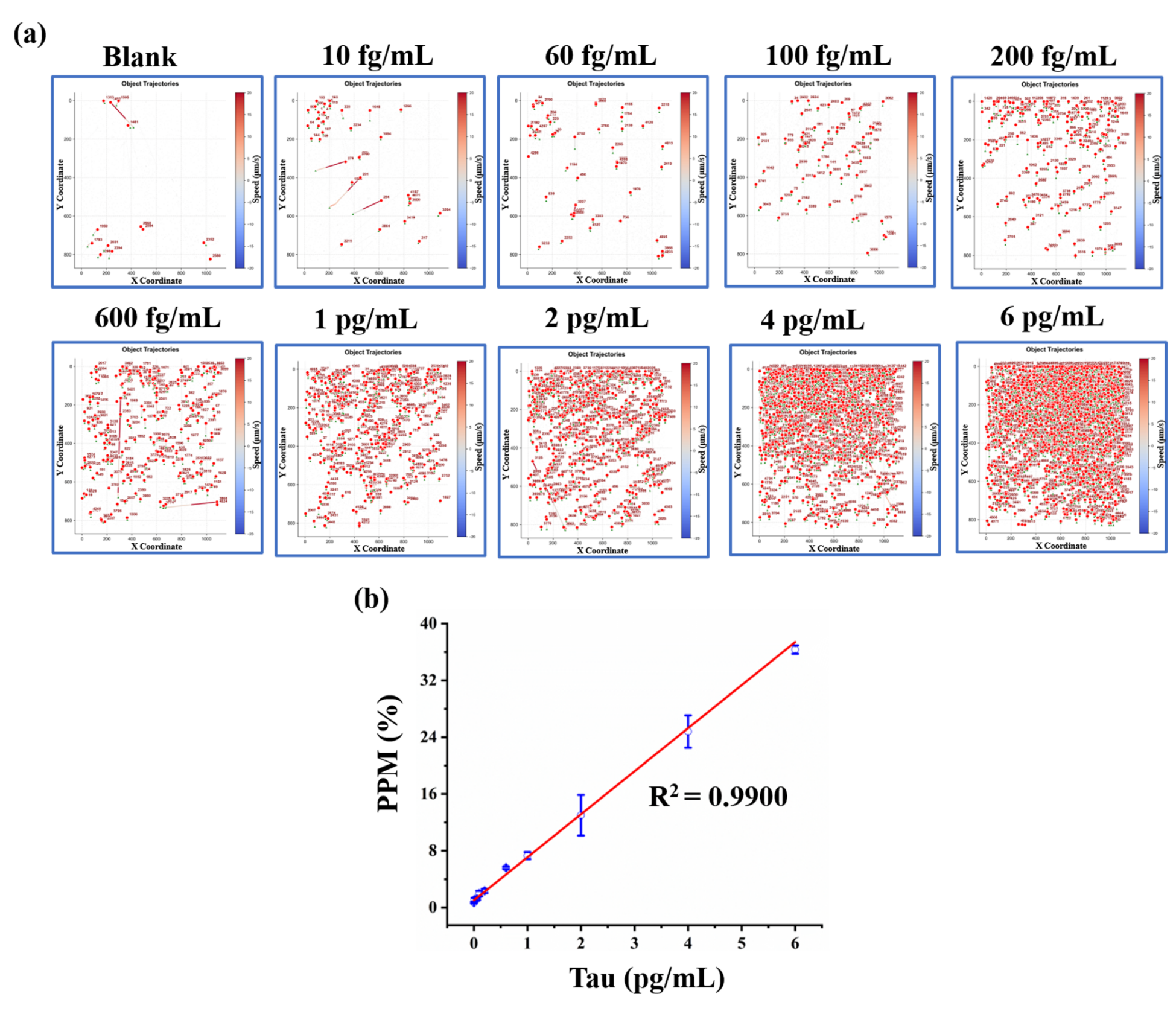
**

**Figure S9.** Exploring the generality of the AI-dMIA for Tau analysis. (a) The number of positive motors induced by different Tau dosages, including 0, 0.01, 0.06, 0.1, 0.2, 0.6, 1, 2, 4, and 6 pg/mL. (b) Linear relationship between the PPM and Tau dosages (0~6 pg/mL). n = 3. Error bars represent the standard deviation from 3 parallel tests.

1. **Evaluating the specificity of the proposed AI-dMIA**

**
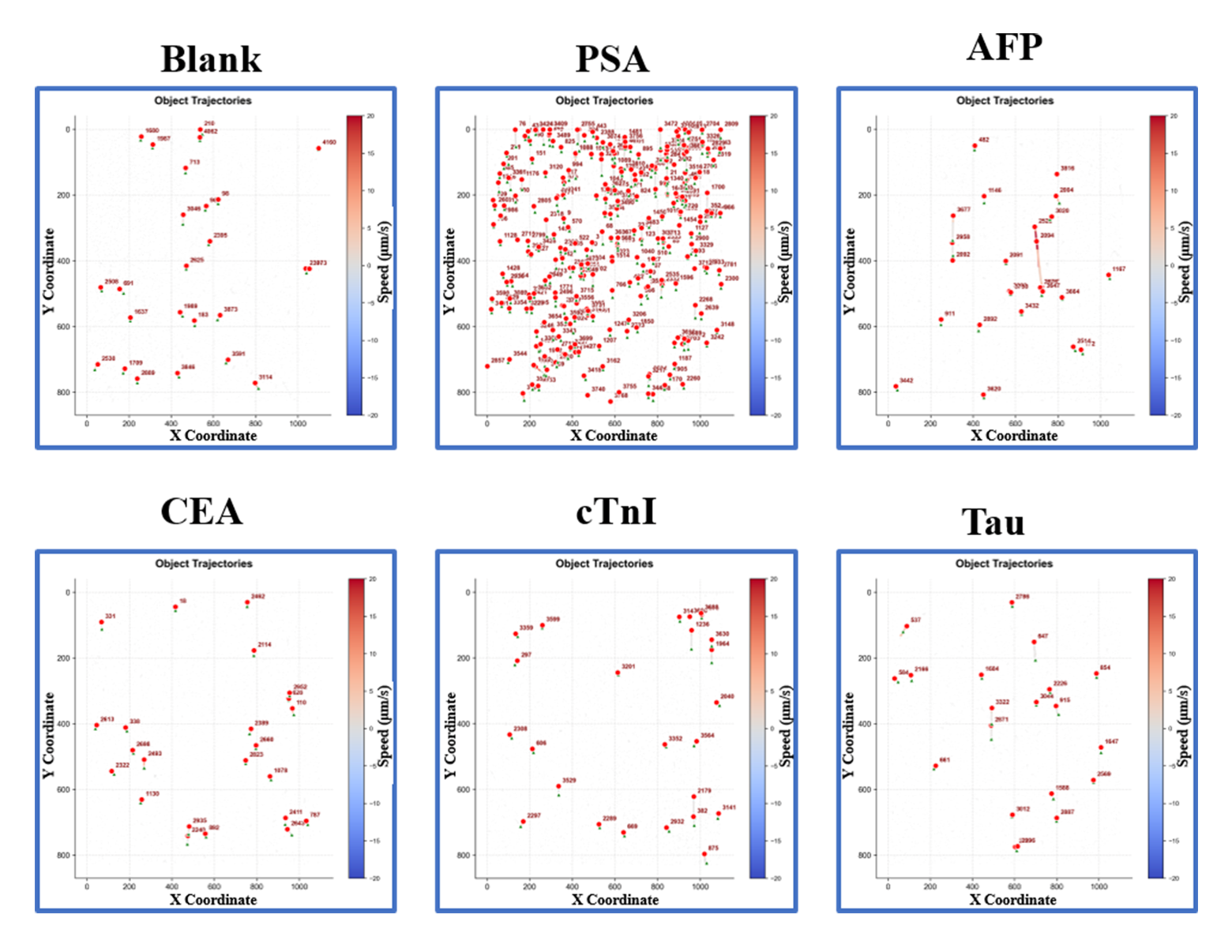
**

**Figure S10.** Evaluating the specificity of the proposed AI-dMIA. The number of positive motors induced by potential interfering proteins, including AFP, CEA, cTnI, and Tau (400 fg/mL for all) by using the PSA-specific antibody pair.

1. **Verification of the specific recognition of PSA by using** **isotype control antibodies**

To verify that the capture of PSA is of specificity, we substituted the PSA-specific antibodies with AFP-specific antibodies to conduct the AI-dMIA. As depicted in Figure S11, when Ab1-PS_6_ (or bio-Ab2) is replaced by Ab1_AFP_-PS_6_ (or bio-Ab2_AFP_) to conduct the AI-dMIA for analyzing PSA, negligible positive motors are observed. Only using PSA-specific antibodies can achieve the specific capture of the PSA molecules, thereby forming positive motors. These results demonstrate that the recognition of PSA molecules is of high specificity.


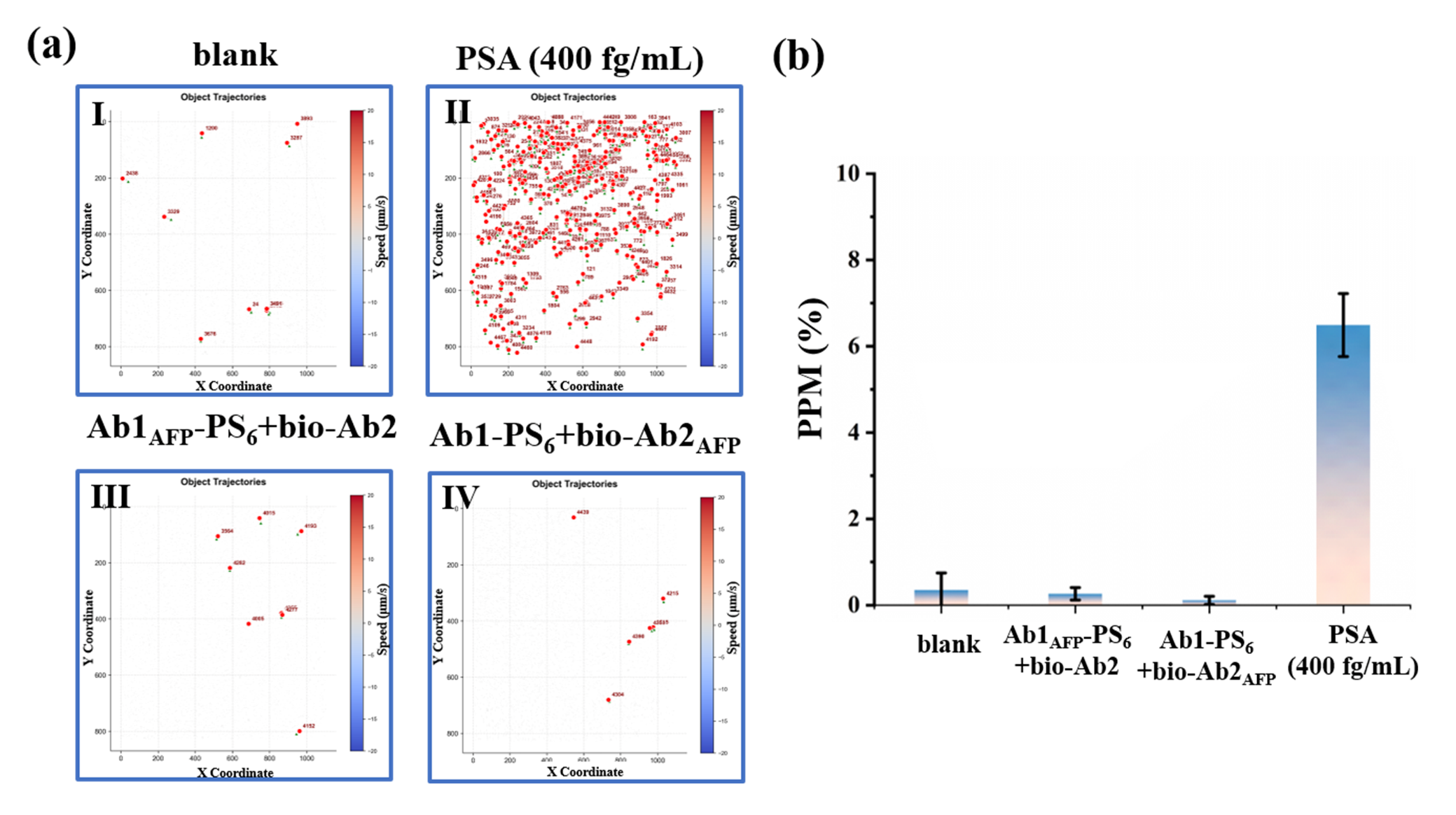


**Figure S11.** Verification of the specific recognition of PSA by using isotype control antibodies. (a) AI-dMIA with different components: AI-dMIA is conducted with the PSA antibody pair in the presence of 0 (I) and 400 fg/mL PSA (II); Ab1-PS_6_ and bio-Ab2 are replaced by AFP-specific antibody-coated PS_6_ (Ab1_AFP_-PS_6_, III) and bio-Ab2_AFP_ (IV). (b) PPMs induced by AI-dMIA under different components. n = 3. Error bars represent the standard deviation from 3 parallel tests.

1. **Detection results of the proposed AI-dMIA by using PS_6_ and MNPs_300_ for PSA analysis**

As the size of MNPs increases, their corresponding magnetic response capacity also enhances. Hence, we wonder if a better digital discrimination between the positive and negative motors in the AI-dMIA can be obtained by substituting MNPs_50_ with MNPs_300_. As shown in Figure S12a, after sandwich immunoreaction and TSA, the MNPs_300_ are introduced to bind to the PS_6_ surfaces to form magnetic motors, which are driven in a magnetic field and tracked by the MTS. As presented in Figure S12b and S12c, as low as 10 fg/mL PSA can be distinguished from the blank control, the performance of which is the same as that caused by MNPs_50_. This may be attributed to the steric hindrance of large particle size, which renders the binding number of MNPs_300_ at the TSA sites limited, thereby failing to improve the digital sensing performance.


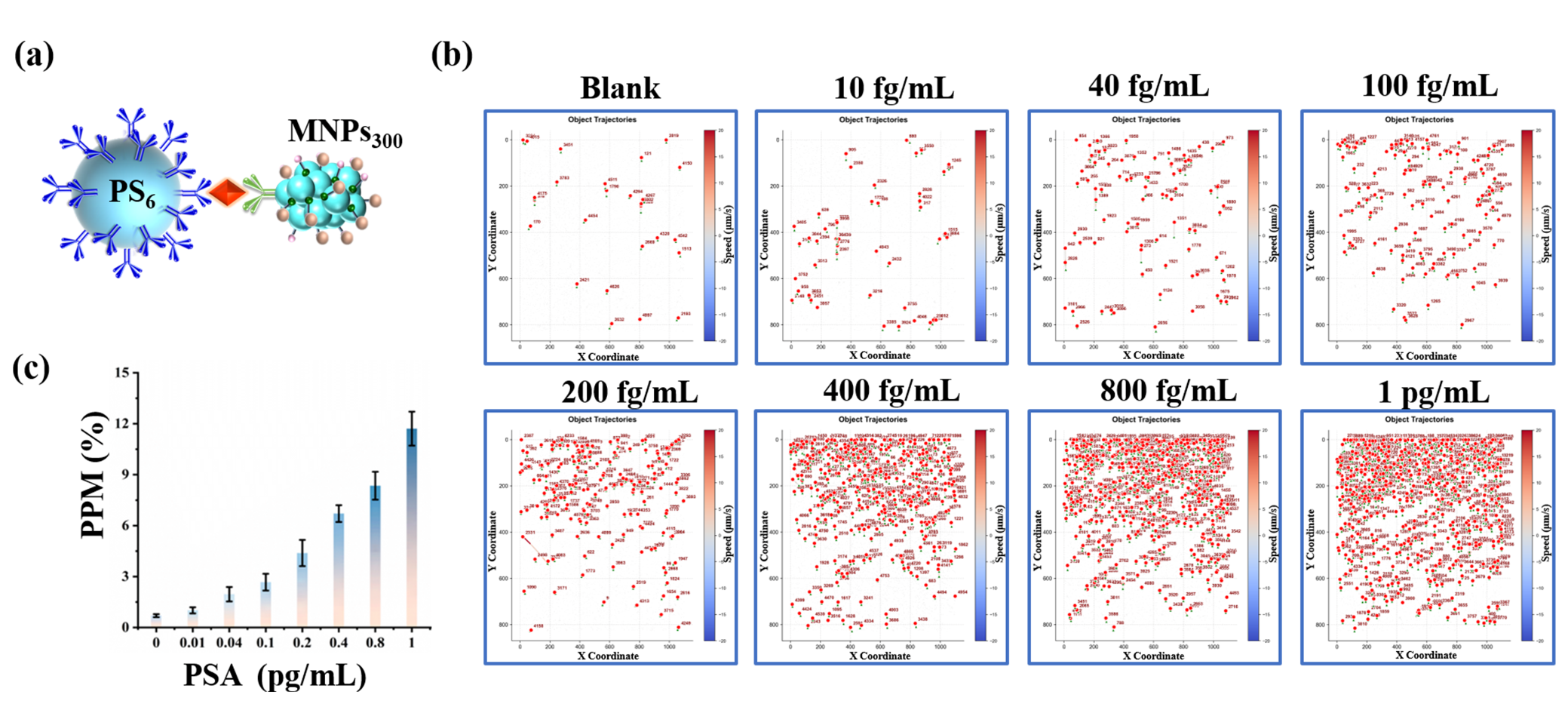


**Figure S12.** (a) Design principle of the proposed AI-dMIA by using PS_6_ and MNPs_300_ for PSA analysis. (b) The number of positive motors induced by different PSA dosages (0, 0.01, 0.04, 0.1, 0.2, 0.4, 0.8 and 1 pg/mL). (c) PPM produced by different PSA concentrations in the range of 0~1 pg/mL. n = 3. Error bars represent the standard deviation from 3 parallel tests.

Movie S1 (separate file). MTS-tracked real-time micromotor trajectories of the blank control by conducting AI-dMIA. (MP4)

Movie S2 (separate file). MTS-tracked real-time micromotor trajectories of the 400 fg/mL PSA by conducting AI-dMIA. (MP4)

Movie S3 (separate file). MTS-tracked real-time micromotor trajectories of the 2 pg/mL PSA by conducting AI-dMIA. (MP4)

Source code: https://github.com/luhuilin/track_ocsort
